# Expression of mutant exon 1 huntingtin fragments in human neural stem cells and neurons causes inclusion formation and mitochondrial dysfunction

**DOI:** 10.1096/fj.201902277RR

**Published:** 2020-04-23

**Authors:** Rhia Ghosh, Alison Wood‐Kaczmar, Lucianne Dobson, Edward J. Smith, Eva C. Sirinathsinghji, Janos Kriston‐Vizi, Iain P. Hargreaves, Robert Heaton, Frank Herrmann, Andrey Y. Abramov, Amanda J. Lam, Simon J. Heales, Robin Ketteler, Gillian P. Bates, Ralph Andre, Sarah J. Tabrizi

**Affiliations:** ^1^ Huntington's Disease Centre Department of Neurodegenerative Disease UCL Queen Square Institute of Neurology University College London London UK; ^2^ MRC Laboratory for Molecular Cell Biology University College London London UK; ^3^ School of Pharmacy Liverpool John Moores University Liverpool UK; ^4^ Evotec AG Hamburg Germany; ^5^ Department of Clinical and Movement Neurosciences UCL Queen Square Institute of Neurology University College London London UK; ^6^ Neurometabolic Unit National Hospital for Neurology and Neurosurgery London UK

**Keywords:** aggregation, Huntington's, mitochondria, model, respiration

## Abstract

Robust cellular models are key in determining pathological mechanisms that lead to neurotoxicity in Huntington's disease (HD) and for high throughput pre‐clinical screening of potential therapeutic compounds. Such models exist but mostly comprise non‐human or non‐neuronal cells that may not recapitulate the correct biochemical milieu involved in pathology. We have developed a new human neuronal cell model of HD, using neural stem cells (ReNcell VM NSCs) stably transduced to express exon 1 huntingtin (HTT) fragments with variable length polyglutamine (polyQ) tracts. Using a system with matched expression levels of exon 1 HTT fragments, we investigated the effect of increasing polyQ repeat length on HTT inclusion formation, location, neuronal survival, and mitochondrial function with a view to creating an in vitro screening platform for therapeutic screening. We found that expression of exon 1 HTT fragments with longer polyQ tracts led to the formation of intra‐nuclear inclusions in a polyQ length‐dependent manner during neurogenesis. There was no overt effect on neuronal viability, but defects of mitochondrial function were found in the pathogenic lines. Thus, we have a human neuronal cell model of HD that may recapitulate some of the earliest stages of HD pathogenesis, namely inclusion formation and mitochondrial dysfunction.

**Abbreviations**
CS: citrate synthase
DD: day of differentiation
FACS: fluorescence‐activated cell sorting
GFP: green fluorescent protein
HD: Huntington's disease
(m)HTT: (mutant) huntingtin
IB: inclusion body
LDH: lactate dehydrogenase
MSNs: medium spiny neurons
NSC: neural stem cell
polyQ: polyglutamine

## 1 INTRODUCTION

Huntington's disease (HD) is an autosomal dominant inherited neurodegenerative disorder, typically of adult onset, with irreversible progression of motor, cognitive, and psychiatric symptoms over 10‐15 years.^1^ There are currently no disease‐modifying therapies. The causative mutation is an expanded CAG repeat in exon 1 of the gene encoding the huntingtin protein (HTT), which leads to an elongated polyglutamine (polyQ) stretch within its N‐terminal domain. The length of the CAG repeat is critical in determining disease status; inheritance of 36‐39 CAG repeats leads to a reduced penetrance form of HD, whereas 40 or more CAGs causes the fully penetrant form. Longer repeat lengths are associated with an earlier age of onset and faster disease progression, with the inheritance of more than 55 CAGs leading to juvenile HD, in which age of onset occurs before 20 years of age.^2^

The “exon 1 fragment” of mutant HTT has long been postulated as a key driver of neurotoxicity in HD. Histopathological studies of post‐mortem human brains reveal that neuronal inclusions are recognized only by N‐terminal HTT antibodies.^3^, ^4^ The R6/2 mouse that expresses a human exon 1 *HTT* transgene has the earliest onset and fastest progressing HD mouse phenotype and has been used extensively in HD research.^5^ A study of N‐terminal fragments in *Drosophila* has confirmed that exon 1 HTT is the most toxic HTT species.^6^ Mutant exon 1 HTT fragments can be generated by an incomplete splicing event resulting in a small exon 1 ‐ intron 1 polyadenylated transcript.^7^ This incompletely spliced transcript is present in all knock‐in HD mouse models^7^ and HD post‐mortem brains and fibroblasts,^8^ supporting a role in HD pathogenesis.

Over‐expression of mutant exon 1 HTT demonstrates toxicity in multiple cell systems,^9^, ^10^, ^11^ but many such cell models are non‐human and/or non‐neuronal and degree of over‐expression of exon 1 HTT is also often unquantified. The recent development of human embryonic and induced pluripotent stem cells has raised the possibility of their use in disease modelling and drug screening, but these models are less amenable to high throughput formats and interpretation of data is frequently confounded by the genetic variability between lines and lack of isogenic controls.^12^ Therefore, we chose to engineer ReNcell VM human neural stem cells (NSCs)^13^ to stably express exon 1 HTT fragments with varying polyQ‐length expansions at relatively matched expression levels upon an isogenic background. ReNcell VM NSCs rapidly differentiate into a high proportion of midbrain GABA‐ergic neurons^13^, ^14^; transduced NSCs maintained exogenous exon 1 HTT expression following differentiation, whilst a small proportion of cells developed mutant HTT‐positive inclusions. High content imaging of this new cell model of HD was carried out using the PerkinElmer Opera platform and mitochondrial respiratory chain function was assessed using the Seahorse XFe^96^ Analyzer and spectrophotometric enzyme assays. This enabled the study of the effect of mutant exon 1 HTT on differentiation and cell metabolism during the switch from NSC to neuronal fate and allowed the evaluation of this model for high throughput drug screening.

## 2 MATERIALS AND METHODS

All reagents obtained from Sigma or Thermo Fisher Scientific unless otherwise stated.

### 2.1 ReNcell VM cell culture

The ReNcell VM NSC line was obtained from ReNeuron. NSCs were grown as a monolayer on Nunc plasticware pre‐coated with laminin [Cultrex mouse laminin I, PathClear, AMS Biotechnology] pre‐diluted 20 µg/mL in cold DMEM‐F12 medium. NSC medium comprised Dulbecco's Modified Eagle's Medium (DMEM): Nutrient Mixture F‐12 (F12) supplemented with: 0.03% of human albumin solution, 5 µg/mL of transferrin (human recombinant), 16.2 µg/mL of putrescine dihydrochloride, 5 μg/mL of insulin (human recombinant), 400 ng/mL of l‐thyroxine, 337 ng/mL of tri‐iodo‐thyronine, 60 ng/mL of progesterone, 2mM l‐glutamine, 40 ng/mL of sodium selenite, 10 Units/mL of heparin sodium, 10 ng/mL of corticosterone, 10 ng/mL of basic fibroblast growth factor [bFGF, Peprotech], and 20 ng/mL of epidermal growth factor [EGF, Peprotech]. NSCs required a full media change every 3‐4 days and were passaged at 80%‐90% confluency. To passage, cells were washed with Hank's balanced salt solution (HBSS) and trypsinized using Trypzean‐EDTA [Lonza] that was added in sufficient volume to coat the surface of the flask and incubated at 37°C; 5% of CO_2_ for 5 minutes. An equivalent volume of defined trypsin inhibitor (DTI) was added to the flask, and the cells were triturated and transferred to a Falcon tube for centrifugation at 350 *g* for 5 minutes at room temperature. The resulting cell pellet was re‐suspended in warm NSC medium prior to re‐seeding at a density of 1.2 × 10^4^ cells/cm^2^. On reaching 90% confluency, ReNcell VM NSCs were switched into differentiation medium (NSC medium without the addition of bFGF and EGF, and supplemented with 2 ng/mL of glial cell‐derived neurotrophic factor [GDNF, Peprotech] and 0.5 mM of dibutyryl cyclic adenosine monophosphate [dbcAMP, Calbiochem]). Differentiating cell cultures had a full media change every 3‐4 days. After two weeks, the differentiation medium was no longer supplemented with GDNF or dbcAMP, and a lower l‐glutamine concentration of 0.5 mM was used. ReNcell VM neurons were maintained in this long‐term differentiation media for up to six weeks, with media changes every 3‐4 days. DD stands for day of differentiation.

### 2.2 Generation of exon 1 HTT expressing ReNcell VM NSCs

ReNcell VM NSCs underwent lentiviral transduction with vectors containing *HTT* exon 1 with 30, 71, and 122 CAG repeats, respectively, each linked with an internal ribosome entry site (IRES) to the gene encoding green fluorescent protein (GFP) (Figure S1A). Sanger sequencing of the constructs was performed to verify sequence fidelity. The *HTT* exon 1 122 CAG stretch contains a CAA interruption at the ninth penultimate codon. The internal ribosome entry site (IRES) sequence allows translation initiation in the middle of an mRNA, allowing for the independent translation of *HTT* exon 1 and *GFP*. The A2UCOE promoter reduces the chance of post‐integration transgene silencing by conferring a dominant chromatin opening function, which is a more transcriptionally active structure.^15^ The woodchuck post‐transcriptional regulatory element (WPRE) is commonly inserted into lentiviral constructs to increase mRNA transport out of the nucleus, hence increasing viral titre. To create a negative control line, ReNcell VM NSCs were transduced with a GFP‐only expressing vector.

For each line, the cells were sorted by fluorescence‐activated cell sorting (FACS) on the basis of GFP expression. Four populations of low to high expressing cells were obtained for each line. Our aim was to find a panel of cell lines with similar expression levels of exon 1 HTT to control for potential expression‐dependent effects of the peptide on cellular phenotype as opposed to increasing polyQ‐repeat length. We chose the lowest expressing lines, which were also most equally matched for protein expression, to use in these experiments. We used a higher expressing 71 CAG line as a positive control (71 CAG‐H) and the non‐transduced wild type ReNcell VM line in some experiments.

### 2.3 Protein detection

Western blotting was carried out using the Li‐Cor system. A methanol precipitation step was included in the preparation of samples for blotting with anti‐HTT antibodies and cell markers. Samples (20 µg protein/sample) were loaded onto Novex 12% of Tris‐Glycine gel (or 8% where stated), with 60‐90 minutes run time at 150 V, followed by overnight transfer step at 15 V. Antibodies used to detect HTT were S830, raised against the product of the N‐terminal region to 53 glutamine residues (sheep, 1:2000^16^) and 4C9; raised against HTT residues 65‐84 (mouse, 1:1000, CHDI Foundation). Other antibodies included anti‐βIII‐tubulin (rabbit, 1:5000, Abcam), anti‐GAPDH (rabbit, 1:10 000, Merck or 1:2500, Abcam), and anti‐nestin MAB5326 (mouse, 1:5000, Millipore). To examine mitochondrial function, we carried out Western blotting for complex subunits NDUFB8 (complex I), SDHB (complex II), UQCRC2 (complex III), MTCO1 (complex IV), and ATP5A (complex V) using the Total OXPHOS rodent WB antibody cocktail at 1:250 (Abcam). To quantify OPA‐1 isoforms, anti‐OPA‐1 antibody (mouse, 1:1000, BD Biosciences) was used. Cell pellets were lysed using RIPA buffer containing complete protease inhibitors and PhosSTOP phosphatase inhibitors (Roche) and 28 U/mL of benzonase. Gel samples were run out on BOLT 4%‐12% of Bis‐Tris Plus gels (Thermo Fisher Scientific) using either MES (OXPHOS) or MOPS (OPA‐1) SDS running buffers. For OXPHOS detection PVDF was used, for all other antibodies nitrocellulose membrane was used. Li‐Cor detection was used to detect bands and densitometry performed using the Image Studio 5.2 software (Li‐Cor).

Cell pellets of each line were made for quantification of total and mutant HTT levels using the Meso Scale Diagnostics ELISA‐based electrochemiluminescence platform.^17^ The 2B7/4C9 antibody pair was used to detect total HTT and 2B7/MW1 antibody pair was used to detect mutant HTT.

### 2.4 Immunofluorescence

Cells were fixed with 4% of paraformaldehyde for 20 minutes at room temperature, and then, washed gently with PBS. Cells were then permeabilized with 0.2% of Triton X‐100 for 15 minutes at room temperature. Blocking was performed by applying 10% of donkey serum diluted in PBS for 1 hour at room temperature. The blocking buffer was removed and primary antibodies pre‐diluted in PBS were applied to cells prior to incubation overnight at 4°C. The primary antibodies used were S830 (sheep, 1:500), EM48 (mouse, Millipore; 1:100), anti‐βIII‐tubulin (rabbit, 1:500, Abcam), and anti‐activated caspase‐3 (rabbit, 1:100, Abcam). The monoclonal antibodies PHP1 and PHP2 (1:1000), raised against the proline‐rich domain (PRD) of HTT were a kind gift from Professor A. Khoshnan.^18^ After five washes in PBS, secondary antibodies were added at a dilution of 1:1000 and incubated for 1 hour in the dark at room temperature. After two further wash steps Hoescht diluted 1 µg/mL in PBS was added for 5 minutes at room temperature. Cells were washed thrice more and stored in PBS containing sodium azide (0.02%) at 4°C in the dark until imaged.

### 2.5 High content imaging

CellCarrier96 plates [Perkin Elmer] were pre‐coated with laminin and seeded with exon 1 HTT over‐expressing ReNcell VM NSCs at a density of 25 000 cells/well. After 24 hours differentiation was initiated. Plates were fixed and stained at various time‐points as described. Image capture for high content imaging was carried out using the PerkinElmer Opera LX or Opera Phenix HCS system using a 40× air NA 0.6 objective, with 15‐18 fields of view captured per well. Channels for Hoechst, 488, 568, and 647 were selected, the power set to 100% and separated, where appropriate to prevent cross‐talk. Heights for imaging were kept constant for each channel and each antibody combination, as crucially, were the gain settings.

### 2.6 Image analysis and statistical evaluation

Image analysis was performed using ImageJ^19^ version 1.50c (IJ) running under Linux on a Tyan FT48‐B8812 High Performance Barebone System equipped with 256GB RAM memory. The open source R software^20^ version 3.2.2 was used for statistical analysis. The results of this are shown in Figure 3. Median of the maximum pixel intensity of nuclei per well (Median I‐max) was calculated as the following: Nuclear channel images were processed with noise reduction by applying a 3 pixel radius median filter followed by segmentation using intensity threshold value 144, which was calculated by applying an Otsu segmentation algorithm on the whole stack of images. Subsequently, a watershed filter was used to separate merged nuclei. The total cell count (tot cells) in each image was calculated from the result and maximum intensity on channel 2 was calculated under the area of each nucleus object. The median of those maximum values was calculated for each well. The total number of aggregate containing nuclei as a proportion of the total cell count (percentage abnormal cells as determined by the total nuclear count) was calculated, where aggregate containing nuclei was defined as nuclei with maximum intensity on ch2 > 2000.

Total number of nuclear aggregates (NAs) per aggregate containing nucleus (ACN, No. NAs per ACN) was calculated with watershed segmentation IJ Find Maxima algorithm using noise parameter 150. Mean background intensity after all aggregates have been removed from the field of view (Mean background) was calculated by segmenting channel 2 images with threshold value 2000 and calculate the mean intensity of pixels less than intensity 2000. Extra‐nuclear aggregate (ENA) count per field of view divided by the total cell count in that field of view (ENAs/tot cells) was calculated using watershed segmentation IJ Find Maxima algorithm with noise parameter 150 applied outside the area of nuclear mask in each image. Total number of aggregates (both intra‐ and extra‐nuclear) per field of view divided by the total cell count in that given field of view (tot aggs/tot cells). Where total number of aggregates was calculated with watershed segmentation IJ Find Maxima algorithm using noise parameter 1000. Total number of nuclear aggregates only per field of view divided by the total cell count in that given field of view (NAs/tot cells) was calculated by the ratio of No. NAs per ACN and tot cells as described above. Total area of nuclear aggregates divided by total nuclear area (NA area/tot nuclear area) was calculated by segmenting the channel 2 area under nuclei with intensity maximum > 2000 with a thresholding value of 969, which resulted in the area of nuclear aggregates in a given nucleus. The total nuclear area was used to calculate this ratio.

**FIGURE 1:**
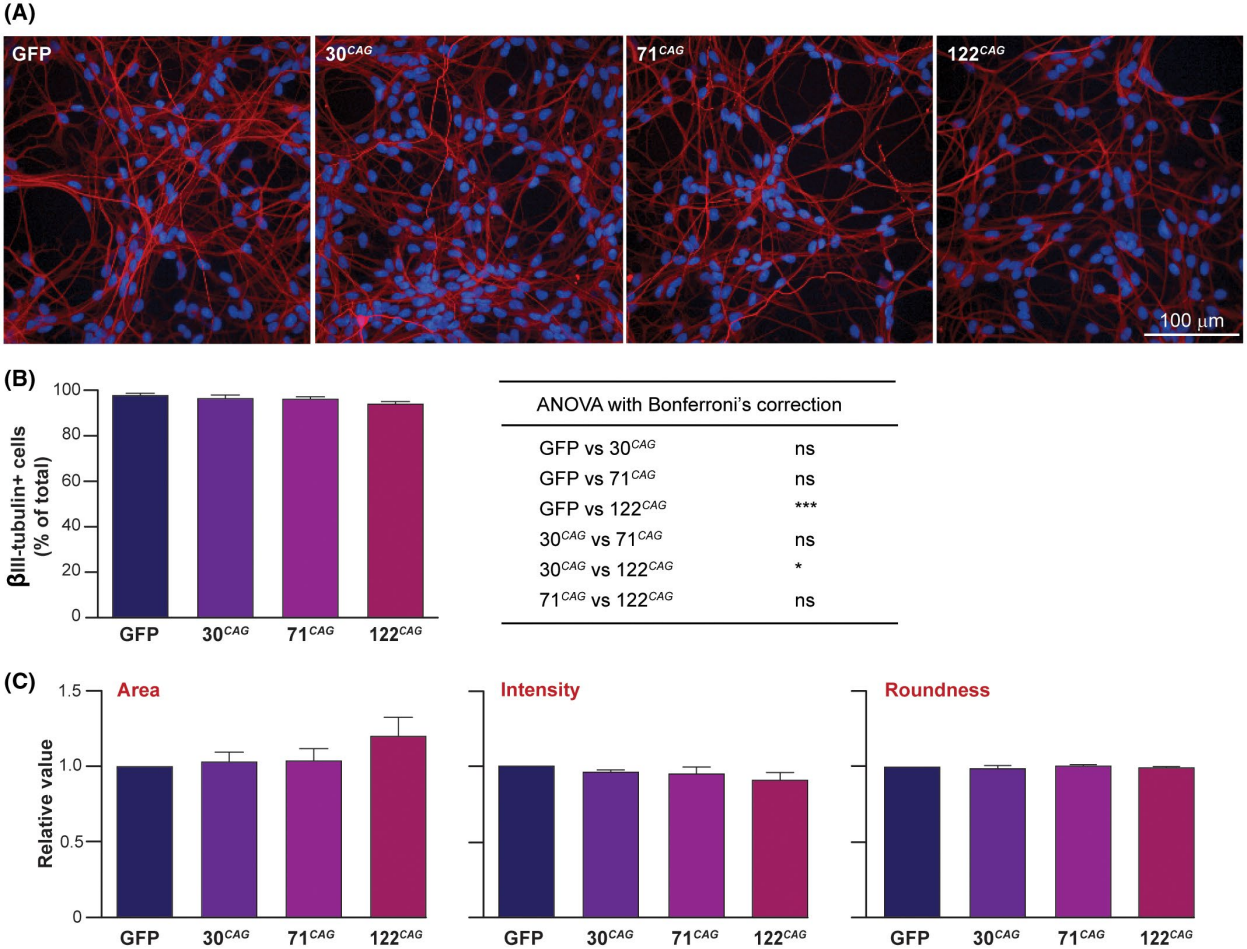
Differentiation of ReNcell VM exon 1 HTT expressing human midbrain NSCs to GABAergic neurons. A, ReNcell VM NSCs that express exon 1 HTT and GFP, as well as GFP‐only cells were differentiated to DD14. Immunofluorescence imaging revealed these cells have a neuronal morphology and express βIII‐tubulin (red, nuclei blue). B, Analysis of βIII‐tubulin staining intensity following high content imaging showed a small but significant decrease in the 122 CAG line compared to the control GFP‐only and 30 CAG lines; one‐way ANOVA with Bonferroni's correction, (n = 8 wells, *P < .05, ***P < .001, ns ‐ not significant). C, Analysis of nuclear metrics using Columbus software showed no significant differences in mean nuclear area, nuclear staining intensity or roundness between any of the lines (values normalized to those of the GFP line); one‐way ANOVA with Bonferroni's correction (n = 80 wells). Data are shown as mean ± SEM

**FIGURE 2:**
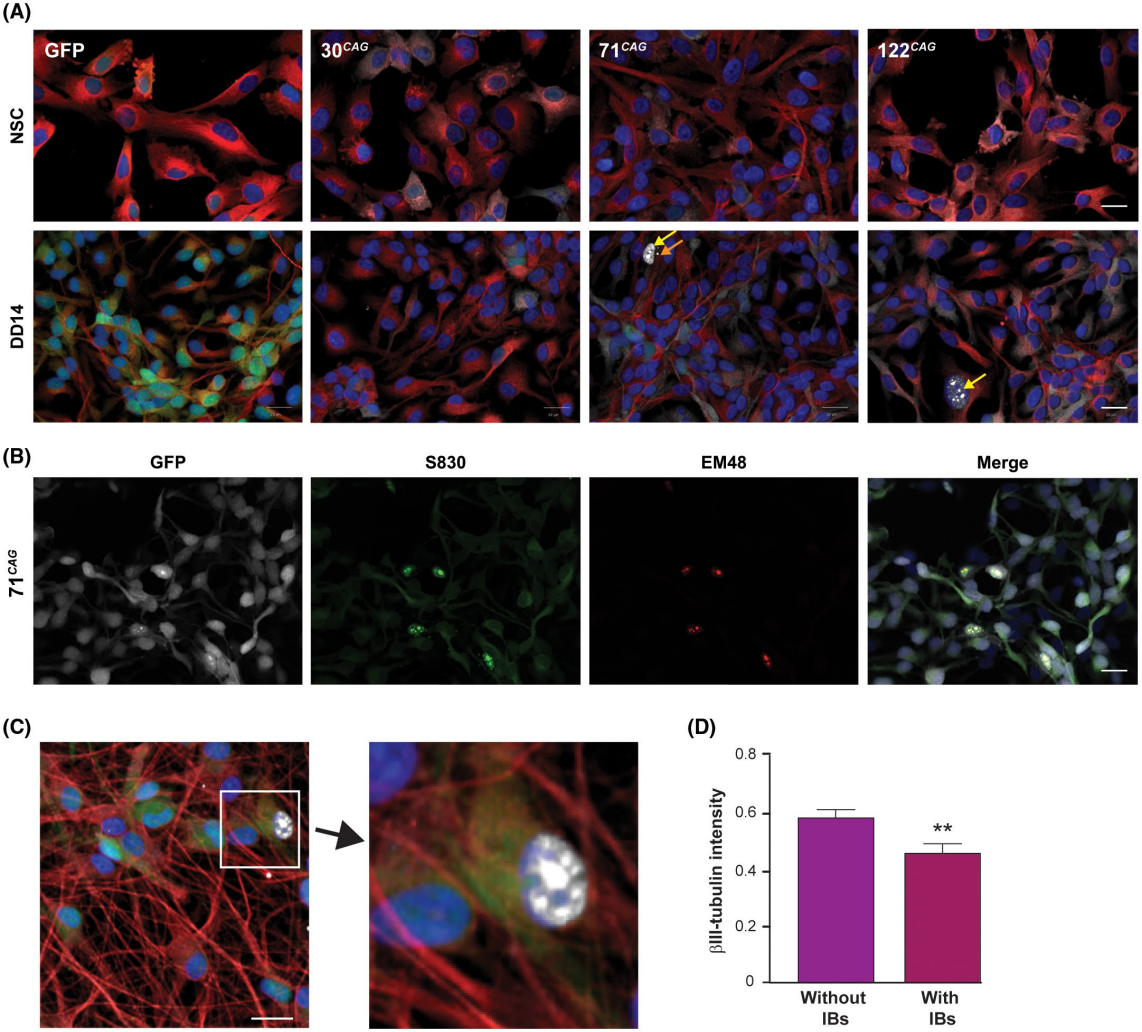
Appearance of mutant HTT inclusions in ReNcell VM exon 1 HTT expressing neurons. A, The exon 1 HTT ReNcell VM lines did not display IB formation in the neural stem cell state. On differentiation to DD14, IBs appeared in the 71 CAG and 122 CAG lines. Intranuclear inclusions shown with yellow arrows, extranuclear inclusions indicated with orange arrows. B, The 71 CAG line with IBs that co‐stain with anti‐aggregated HTT antibodies S830 (green) and EM48 (red). GFP is shown in white. C, Nuclei that contained inclusions had reduced staining intensity in the cytoplasm, an example of which has been enlarged (red ‐ βIII‐tubulin; white ‐ anti‐HTT antibody S830; blue ‐ nuclei). Scale bars = 20 µm. D, Quantification of this phenomenon confirmed that mean βIII‐tubulin intensity in IB‐containing cells was significantly less than in non‐IB containing cells (n = 32 wells, **P < .01). Data are shown as mean ± SEM

Images captured by the Opera Phenix were analyzed using Perkin Elmer Columbus v. 2.8.0 image analysis software. In summary, individual planes were analyzed (without flatfield correction). Nuclei were identified using the software's Find Nuclei function and the area, intensity, and roundness of Hoechst staining was measured. The total nuclear count was used as a surrogate marker for the total cell count in each image. βIII‐tubulin + cells were detected and counted using the Find Cytoplasm function, the mean intensity of staining measured and the neuronal population selected by thresholding on mean intensity. Nuclear inclusion bodies (IB) were identified using the Find Spots function using Method B (detection sensitivity 0.1, splitting co‐efficient 0.5). Appropriate population outputs and formula outputs were selected for Define Results, and the data downloaded and exported for statistical analysis using GraphPad Prism 6. Data were analyzed as the mean of one well of each cell line and condition, subject to one‐way or two‐way ANOVA with Bonferroni's correction. A 95% confidence interval (*P* < .05) was considered a statistically significant observation. The results of this are shown in Figures 1B,C, 2D, and 4B.

### 2.7 Structured illumination microscopy

Super resolution fluorescent images were captured using a Nikon Eclipse Ni‐E N‐ SIM Super Resolution system and Andor Ixon camera. Images were saved as ND2 files and processed using NIS Elements AR software with n‐SIM module. Final images were exported as TIFF file images. Acquisition was performed in a 3D SIM mode using the 100 × 1.49 NA total internal reflection fluorescence objective lens with 28 z steps per stack at a step interval of 0.1 µm.

### 2.8 Viability and ATP assays

Clear plastic 96 well plates [Nunc] were pre‐coated with laminin and seeded with exon 1 HTT expressing ReNcell VM NSCs at a density of 25 000 cells/well. After 24 hours differentiation was initiated. Cell death and viability of cultures was assessed at regular time points using a lactate dehydrogenase (LDH) release assay (CytoTox 96 Non‐Radioactive Cytotoxicity Assay [Promega]) or MTT assay (In Vitro Toxicology Assay Kit, MTT based [Merck]) as per the manufacturer's instructions. ATP levels were quantified using Promega's CellTiter‐Glo 2.0 Assay according to the manufacturer's instructions, using six biological replicates per experimental line/condition. For NSCs, cells were dissociated using Accutase (Thermo Fisher Scientific) and 27 000 cells/reaction were used. Neurons cultured in 96 well plates were first dissociated by incubating in 100 µL/well Accutase for 15 minutes, then, gently dissociating by trituration. Next, 90 µL of this was added to the 1× reaction mixture, and 10 µL used to count cells. Luminescence readings per well were then normalized to input cell number. CoQ_10_ used in supplementation experiments was purchased from Sigma and dissolved in ethanol.

### 2.9 Seahorse XFe^96^ bioanalyser

NSCs were plated into laminin‐coated (20 µg/mL) Seahorse XF Cell Culture PS Microplates (Agilent) at 30,000 per well in NSC medium. NSC measurements were performed the following day. For neuronal measurements, the cells were switched into 200 µL differentiation medium per well after 24 hours. After 5 days differentiation, all media was removed and replaced a final time with fresh differentiation medium. The Seahorse XF Cell Mito Stress Test was performed at DD7 due to the fragility of the neurons in this assay at DD14. Seahorse Bioanalyser XFe^96^ assays were carried out according to the manufacturer's instructions (Agilent). The XF assay medium was made up by supplementing XF base medium (Agilent) with 10 mM of d‐glucose, 2 mM of glutamine, 1 mM of sodium pyruvate, and adjusted to pH 7.4. The neurons were washed gently twice with warm XF assay medium before replacing with 175 µL final volume medium. Cells were equilibrated prior to the assay by placing in a 37°C CO_2_‐free incubator for 20 minutes. The test compound concentrations were determined by separate optimization experiments for either NSCs or neurons and set as follows: Oligomycin (1.0 µM), FCCP (2 µM for NSCs, 0.2 µM for neurons), and rotenone/antimycin A (2 µM). For more robust NSC assays, the assay cycles were set at mix 40 seconds, wait 30 seconds, measure 2 minutes. For the more fragile neurons, the assay cycles were set at mix 10 seconds, wait 30 seconds, measure 2 minutes. Following each assay, microplates were retained, and cells fixed with 10% of formalin (Sigma) at room temperature for 15 minutes, before staining with DAPI and imaging on a MetaXpress plate reader. Mean cell densities/well were calculated using ImageXpress and each well density was normalized to the lowest density value. Individual well OCR measurements (pmol/min) for each time point were then divided through by the normalized well cell density value for that well, using the normalization function of the Seahorse Wave Desktop software (Agilent). Respiratory parameters were calculated using this software, as per the manufacturer's instructions. Non‐mitochondrial respiration = (minimum rate measurement after rotenone/antimycin A injection), basal respiration = (last rate measurement before first injection) − (non‐mitochondrial respiration), maximal respiration = (maximum rate measurement after FCCP injection) − (non‐mitochondrial respiration), proton leak = (minimum rate measurement after oligomycin injection) − (non‐mitochondrial respiration), ATP production = (last rate before oligomycin injection) − (minimum rate measurement after oligomycin injection), coupling efficiency = (ATP production rate)/(basal respiration) × 100. Mean parameters for each line were calculated from 10 technical replicates and further normalized as a percentage of the 30 CAG line mean. In initial experiments, the 30 CAG line was found to be phenotypically indistinguishable from the GFP only line so 30 CAG was used as the experimental control line for these assays. The assays were repeated three times and data compared using one‐way ANOVA with Tukey's multiple parameter test.

### 2.10 Mitochondrial respiratory chain activity

Activities of respiratory chain complex I ([NADH dehydrogenase, EC 1.6.5.3]; nmol/min/mg total protein), complex II + III ([succinate: cytochrome c reductase]; nmol/min/mg total protein), complex IV ([cytochrome c oxidase: EC 1.9.3.1]; k/min/mg total protein), and citrate synthase ([CS, EC 2.3.3.1]; nmol/min/mg total protein) were determined in the differentiated ReNcell VM neurons as previously described.^21^ The principal of the complex II + III assay is that succinate is oxidized by complex II and these electrons are then transferred from complex II to complex III by endogenous CoQ_10_. These electrons can be used by complex III to reduce oxidized cytochrome *c*. In the assay, complex II + III activity is then measured by the succinate‐dependent antimycin A‐sensitive reduction of cytochrome *c,* which is followed at 550 nm according to the method of King.^22^ Statistical analysis: Repeated measures ANOVA with Tukey post hoc test. Complex II activity assay was modified from Hatefi and Stiggall.^23^ Complex II‐specific activity (nmol/min/mg total protein) was measured at 30°C following the reduction of 2,6‐dichloroindophenol (DCPIP) at 600 nm (molar ε = 21 000 M‐1 cm^−1^). Cellular homogenates were pre‐incubated for 10 minutes a cuvette containing 50 mM of potassium phosphate buffer pH 7.4, 20 mM of sodium succinate, 0.1 mM of di‐potassium EDTA, 74 µM of DCPIP, 1 mM of potassium cyanide, 10 µM of rotenone. The reaction was started with 50 µM of ubiquinone. The reaction was monitored for 5 minutes after which 1 mM of 2‐thenoyltrifluoroacetone (TTFA) to the cuvette and the reaction monitored for a further 10 minutes. The enzyme rate was determined to be the TTFA sensitive reduction of DCPIP. Complex III activity assay was modified from Kirby et al.^24^ Complex III‐specific activity (k/min/mg total protein) was measured at 30°C following the reduction of cytochrome c using ubiquinol‐2 as the electron donor and monitoring the increase in reduced cytochrome c at 550 nm (molar ε = 19.2 M^−1^ cm^−1^). A 1 mL cuvette containing 35 mM of potassium phosphate buffer pH 7.2, 5 mM of magnesium chloride, 1 mM of di‐potassium EDTA, pH 7.2, 2 mM of potassium cyanide, 15 µM of cytochrome c, 5 µM of rotenone was pre‐incubated for 2 minutes. Non‐enzymatic reduction of cytochrome c was initiated with the addition of 15 µM of ubiquinol‐2 and monitored for 2 minutes, after which cellular homogenate was added and the reaction monitored for 2 minutes. As the enzyme reaction rate is non‐linear, activity was expressed as pseudo first order rate constant k after subtraction of the non‐enzymatic rate. The CoQ_10_ status (pmol/mg total protein) of cell samples was determined by reverse phase HPLC with UV detection at 275 nm according to the method of Duncan et al, 2005.^25^ Readouts are calculated as ratios of complex activity:CS activity or CoQ_10_ status:CS activity to account for variation in mitochondrial content.^26^ Statistical analyses: Student's *t* test between three independent 30 CAG and 122 CAG DD14 neuronal cultures.

### 2.11 TMRM and volumetric analysis

Basal mitochondrial membrane potential was analyzed using TMRM as described previously.^14^ Individual TMRM + mitochondria detected within cells using Image J were classified as small (<100 µm^3^), medium (100‐1000 µm^3^), or large (>1000 µm^3^), and the sum of all mitochondrial volumes within each category expressed as a percentage of total mitochondrial volume.

## 3 RESULTS

### 3.1 Generation of NSCs expressing exon 1 HTT with increasing CAG repeat lengths

ReNcell VM NSCs were transduced with lentiviral vectors containing *HTT* exon 1 with pathological 71 and 122 CAG repeats, and also a non‐pathological 30 CAG repeat length, each linked with an internal ribosome entry site (IRES) to the gene encoding GFP (Figure S1A)*.* To create a negative control line, ReNcell VM NSCs were transduced with a *GFP*‐only expressing vector. The cells were then sorted by FACS on the basis of GFP expression, creating populations of cells with equivalent expression levels. The GFP expression in the NSCs is shown in Figure S1D. Expression of exon 1 HTT was confirmed in NSCs and differentiated neurons and precise protein levels were quantified using a Meso Scale Diagnostics ELISA‐based assay with 2B7 and 4C9 anti‐HTT antibodies (Figure S1B). Western blotting cell lysates from each line using S830 and 4C9 antibodies confirmed robust expression of exon 1 HTT (Figure S1C). Each CAG repeat sequence is followed by CAA CAG codons that are also translated as glutamine (Q) residues; thus, an allelic series with closely matched, relatively low expression levels of 32Q, 73Q, and 124Q exon 1 HTT was established.

### 3.2 Neural stem cells expressing exon 1 HTT can be differentiated into neurons that develop intra‐ and extra‐ nuclear inclusions

The ReNCell line expresses relevant neuronal markers following differentiation, including βIII‐tubulin, MAP2, GABA, tau proteins, and others.^13^, ^14^, ^27^ Upon initiation of differentiation and subsequent culture for fourteen days (DD14), the ReNcell VM exon 1 HTT lines also generated a high proportion of neurons, confirmed using immunofluorescence and Western blotting with anti‐βIII‐tubulin antibodies (Figure 1A and Figure S2A). Quantification of images using high volume image data analysis software (PerkinElmer Columbus) confirmed that 96%‐97% of cells expressed the neuronal marker βIII‐tubulin, except for the 122 CAG line, which had a slightly lower proportion at 94% (Figure 1B). However, no significant difference in overall βIII‐tubulin protein level was observed in this line by Western blotting (Figure S2B,C). Analysis of nuclear metrics by immunofluorescence (mean area, intensity of Hoechst staining, and roundness) across the panel of cells showed no differences between the lines (Figure 1C).

We did not detect extra‐ or intra nuclear HTT inclusions in NSCs in any lines (Figure 2A). However, following differentiation to neuronal cultures (DD14), the pathogenic lines (71 CAG and 122 CAG) were found to harbour intra‐nuclear IBs and a small number of extra‐nuclear IBs, as detected by immunofluorescence (Figure 2A). These inclusions were found to stain with the polyclonal S830 and monoclonal EM48, PHP1, and PHP2 anti‐HTT antibodies (Figure 2B and Figure S2D). IBs were not present in the GFP only control or 30 CAG line. Western blotting using the same antibodies detected high molecular weight bands retained within the stacking gel following SDS‐PAGE; confirming the presence of insoluble forms of mutant HTT exon 1 in the 71 and 122 CAG lines only (Figure S1C). Interestingly, we observed that cells containing IBs appeared to have lower or absent βIII‐tubulin staining (Figure 2C); quantification of this using Columbus software confirmed that there was a significant reduction in the perinuclear cytoplasmic staining intensity, where IBs were present (Figure 2D).

### 3.3 Inclusion body formation increases over time and displays CAG repeat‐length dependence

**FIGURE 3:**
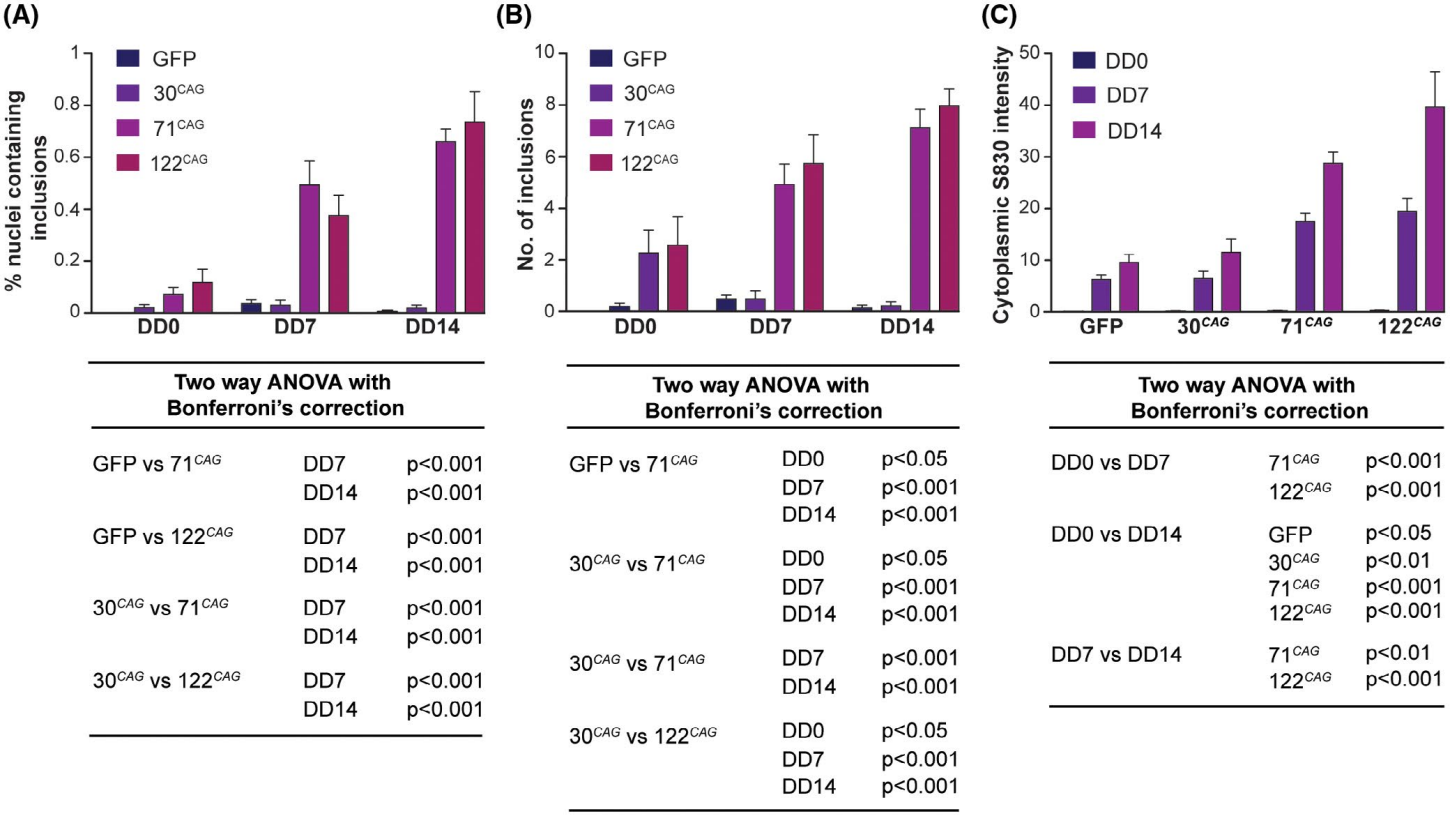
Accumulation of HTT inclusion bodies in ReNcell VM neurons in a time and exon 1 HTT CAG‐dependent manner. High content imaging of the ReNcell VM exon 1 HTT lines at different stages of differentiation revealed that: A, The percentage of cells with nuclear IBs was CAG‐length‐dependent, with nuclear IBs appearing only in the 71 CAG and 122 CAG lines after DD7. B, The number of inclusions per inclusion containing nucleus was also CAG‐length‐dependent. C, The diffuse cytoplasmic anti‐HTT (S830) antibody staining also increases over time compared with non‐HD lines. Data are shown as mean ± SEM. Corresponding significance levels calculated using two‐way ANOVA with Bonferroni's correction are shown below each graph (n = 16 wells per line)

Using Image J, we were able to employ high throughput analysis techniques to quantify the intra‐nuclear S830‐positive IBs and diffuse cytoplasmic staining observed in our images (Figure S2E). We were unable to accurately quantify extra‐nuclear IBs using Image J and R due to their paucity and small size. Significant CAG repeat length‐dependent differences in the percentage of IB‐containing nuclei appeared over time (Figure 3A), with no significant change in total cell count over time (Figure S3A). The number of inclusions per inclusion‐containing nucleus also increased on differentiation and was CAG‐repeat length dependent (Figure 3B). We also detected diffuse cytoplasmic S830 staining in the exon 1 HTT lines, which increased with time above background levels in the 71 and 122 CAG lines (Figure 3C).

**FIGURE 4:**
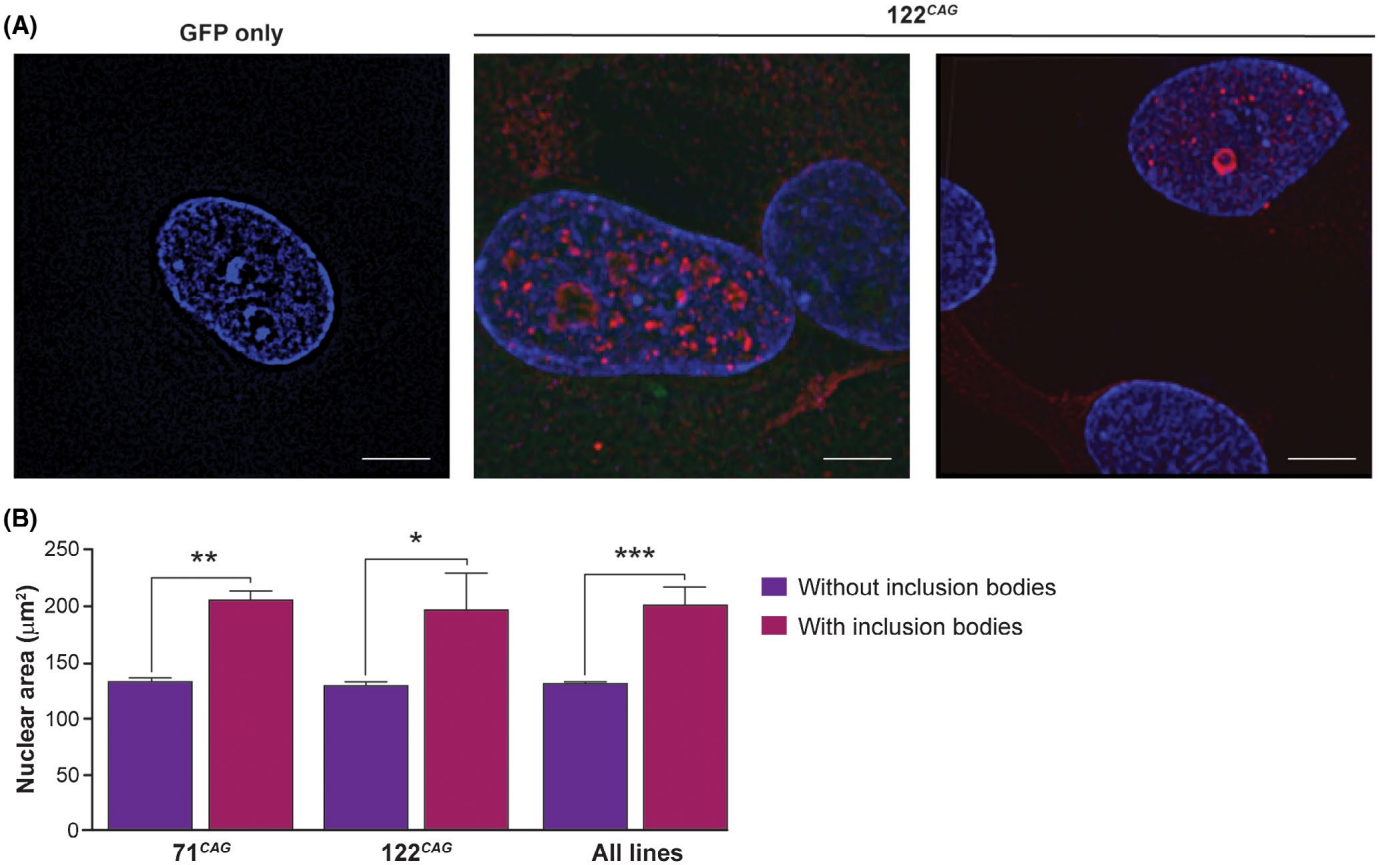
Structured illumination microscopy images of ReNcell VM exon 1 HTT neurons. A, Super‐resolution images of fixed and stained 122 CAG ReNcell VM neurons demonstrated that multiple smaller nuclear inclusions not previously seen with standard confocal microscopy can be seen. The GFP only line had no detectable S830 staining. Blue ‐ Hoescht; red ‐ anti‐HTT antibody S830. Scale bars = 5 µm. B, Quantification of nuclear area from differentiated cultures showed mean nuclear area is greater in cells containing IBs. Data are shown as mean ± SEM. Statistics: one‐way ANOVA with Bonferroni's correction. (n = 32 wells, *P < .05, **P < .01, ***P < .001)

We further explored IB formation using structured illumination microscopy (SIM), with a resolution of 100‐120 nm. This revealed multiple smaller nuclear inclusions previously unseen with standard confocal microscopy (Figure 4A). We also observed the fine diffuse granular cytoplasmic staining of S830. No nuclear or cytoplasmic S830 staining at all was detected in GFP‐only cells suggesting this is not a staining artefact. Overall, nuclear morphology was not observed to be compromised by the presence of inclusions, and the presence of nuclear IBs did not coincide with nuclear fragmentation or condensation that would be indicative of cell death. However, upon quantification, we found that mean nuclear size was significantly increased in IB containing cells compared to non‐IB containing cells (Figure 4B). Unfortunately, due to the overlapping and complex nature of neuronal cultures, it was impossible to assess whether IB‐containing neurons had also an increased overall cell size.

### 3.4 Mutant exon 1 HTT expression has no effect on baseline cell viability

We assessed potential pathology‐associated differences in baseline viability in the exon 1 HTT ReNcell VM panel by performing LDH assays at weekly intervals from DD0 ‐ DD42. This finding was also confirmed using MTT assay, as well as immunofluorescence staining using anti‐activated caspase 3 antibody. No significant differences in baseline viability between the lines were observed (Figure S3).

### 3.5 Mutant exon 1 HTT expression causes respiratory chain deficits

**FIGURE 5:**
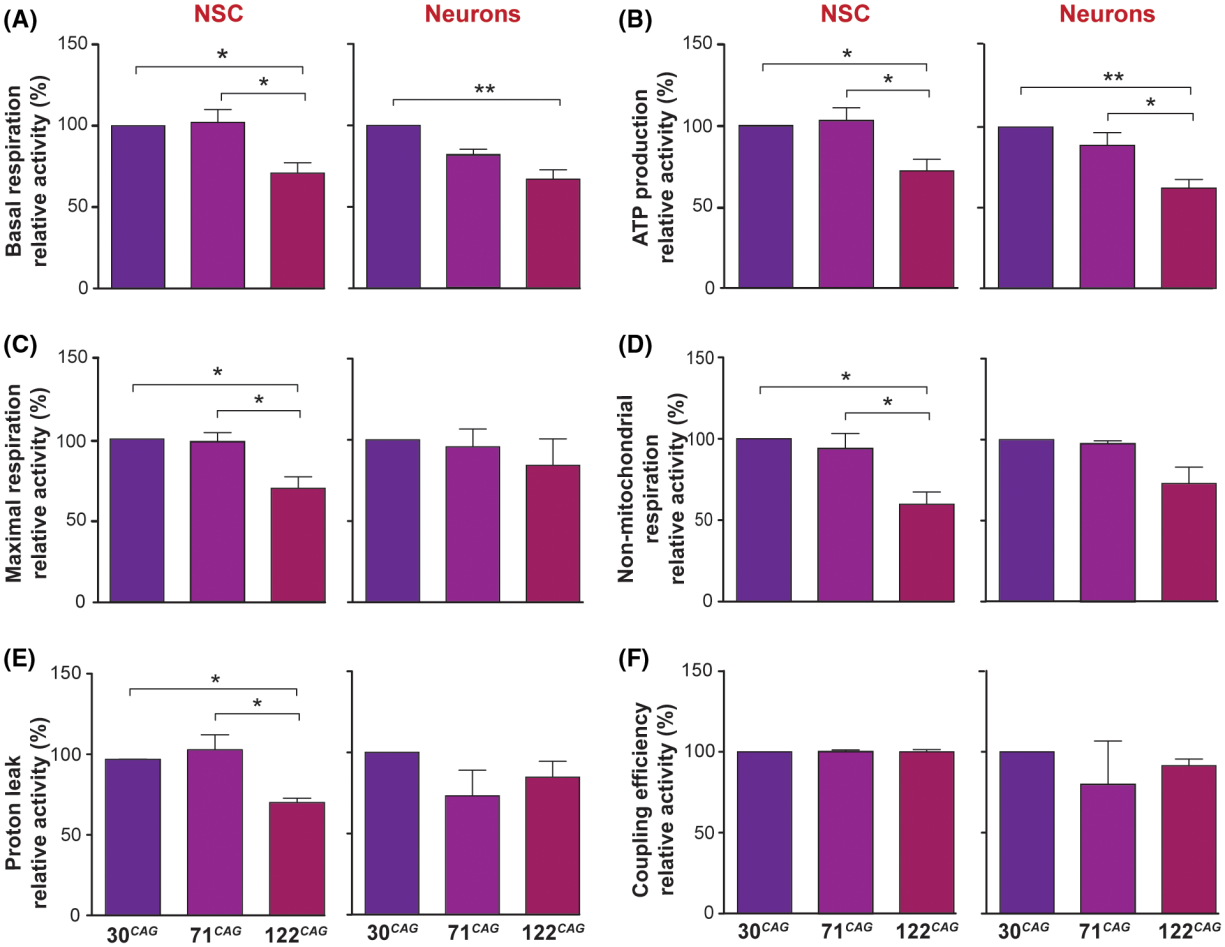
Mitochondrial respiratory function is compromised in the ReNcell VM 122 CAG line. The following respiratory parameters were calculated from OCR measurements obtained during Seahorse XF Mito Stress Tests for both NSC and DD7 neurons: A, Basal respiration. B, ATP production. C, Maximal respiration. D, Non‐mitochondrial respiration. E, Proton leak, and F, Coupling efficiency. Means were taken from three independent experiments (10 technical replicates per line, per experiment) and normalized to the 30 CAG control line readouts, (*P < .05, **P < .01). Data are shown as mean ± SEM

Despite no overt loss of viability, oxidative metabolism was significantly altered in a CAG length‐dependent manner in NSCs and young neurons (at DD7) as measured using a Seahorse XF Cell Mito Stress Test assay. There was a profound reduction in basal respiration in the 122 CAG line even at the NSC stage and a trend to worsening of basal respiration in the 71 CAG line following differentiation and the appearance of mutant HTT inclusions (Figure 5A). Crucially, ATP production ‐ inferred from the oligomycin‐sensitive component of OCR ‐ was significantly reduced in the 122 CAG line at both developmental stages, implicating a reduction in basal ATP production in the presence of mutant exon 1 HTT (Figure 5B). This reduction in total ATP levels was confirmed in 30 CAG and 122 CAG NSCs and neurons using the microplate CellTiter‐Glo 2.0 Assay (Figure S4A). In addition, a significant decrease in maximal respiration rate ‐ assessed by the addition of the de‐coupler FCCP ‐ was observed in the 122 CAG line (Figure 5C) in NSCs but not following differentiation. This corrective phenotype was seen in the non‐mitochondrial component of OCR, which is the minor proportion of oxygen consumption attributable to non‐ETC enzymes. The same phenomenon was observed with proton leak, which can be a sign of mitochondrial damage; although significantly altered in the 122 CAG line NSCs, this was not impaired in neurons (Figure 5D,E). There were no differences in coupling efficiency between the lines in either their NSC or neuronal state (Figure 5F).

**FIGURE 6:**
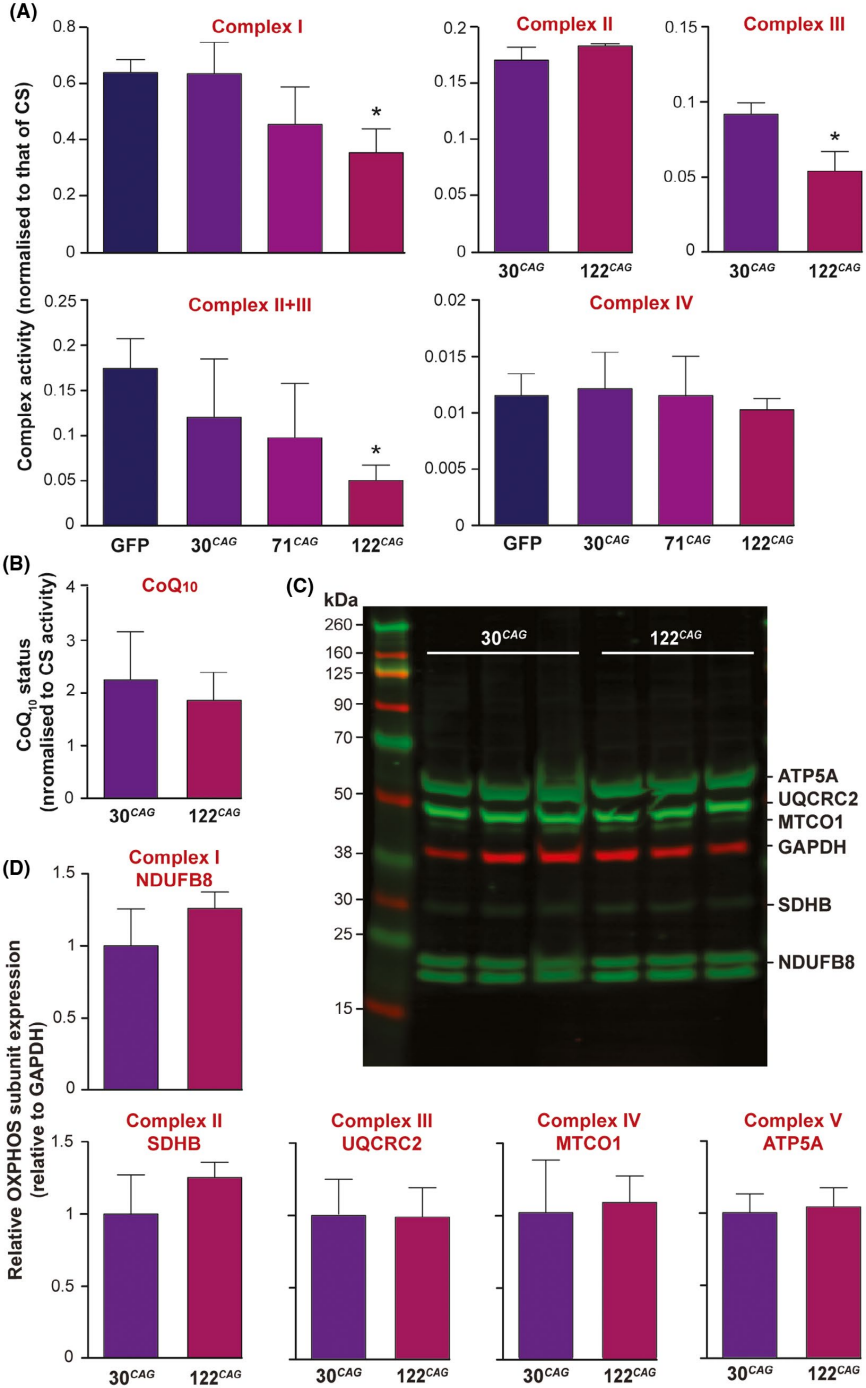
Electron transport chain defects in ReNcell VM 122 CAG neurons. A, The activities of complex I and II + III are significantly reduced in the 122 CAG line (*P < .05) but complex IV was unaffected compared across all lines at DD14. To further pinpoint the defect in mitochondrial respiration, the 30 CAG and 122 CAG lines were assayed for complex II and III and B, CoQ_10_ status. CoQ_10_ status is expressed as pmol/mg divided by CS activity. No significant difference was found in complex II activity or the electron carrier CoQ_10_, but a significant reduction in complex III activity was detected in the 122 CAG neurons (*P < .05). All complex activities are normalized to CS activity to correct for mitochondrial enrichment of the samples, and normalized for total protein. Means were calculated for three samples generated from three independent differentiations per line. Data are shown as mean ± SEM. C, The relative levels of mitochondrial complex subunits were measured by Western blotting of labile subunits from complexes I‐V (green) vs GAPDH (red). D, Quantification of expression levels of individual complex subunits shown in C by band densitometry, normalized to GAPDH. No significant differences in expression were found. Data are shown as mean ± SEM

To examine the underlying mechanism for the respiratory impairments observed in neurons, we assessed the specific in vitro activities of electron chain components using spectrophotometric enzyme assays. Corresponding impairment in the specific activities complex I and II + III but not in complex IV in 122 CAG lines were observed when comparing across the whole panel of exon 1 HTT lines at DD14 (Figure 6A), in line with many previous reports.^28^ Upon finding a deficit in complex II + III activity, we devised assays to measure complex II and III individually, as well as total CoQ_10_ status, which is required for complex II + III activity.^29^ We compared 30 CAG vs 122 CAG neurons and found that although complex II activity did not differ between the lines, complex III activity was significantly reduced in the 122 CAG neuronal samples (Figure 6A). We found no decrease in the levels of total CoQ_10_ between the lines, suggesting that complex III activity was not being compromised by reduced CoQ_10_ availability (Figure 6B).

We wished to examine whether this activity impairment was caused by a reduction in complex expression, so performed Western blots using antibodies against complex subunits NDUFB8 (complex I), SDHB (complex II), UQCRC2 (complex III), MTCO1 (complex IV), and ATP5A (complex V) (Figure 6C). These subunits were chosen because they are labile when not assembled within a complex, therefore, can be used as a proxy readout of expression for each complex. No significant differences were found in the expression of any of these subunits, when normalized to GAPDH (Figure 6D), suggesting that impairment in complex I and III activity is not caused by reduced expression of these complexes. To investigate a potential link between mitochondrial respiratory dysfunction and intranuclear mHTT IB formation in our 122 CAG neurons, we increased endogenous ATP levels by supplementing differentiating neurons with 5 µm CoQ_10_ ^30^ in the culture medium from DD7. Interestingly, this had no impact on the frequency of nuclei containing S830 + IBs by DD14 (Figure S4B), suggesting that restoring ATP production does not directly affect IB formation.

**FIGURE 7:**
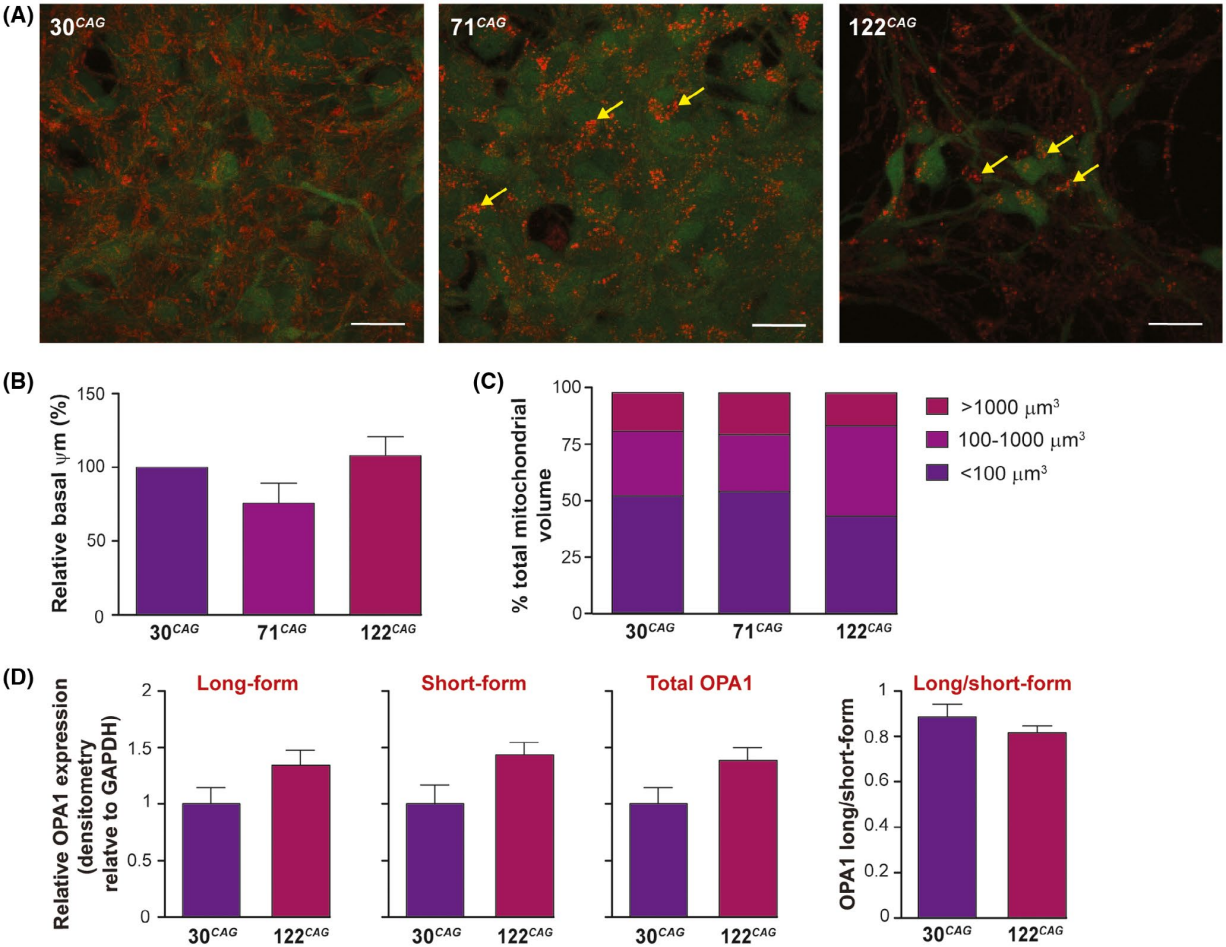
Mitochondrial morphology in ReNcell VM 122 CAG neurons. A, Maximum intensity projection images of z‐stacks taken during live imaging of exon 1 HTT neurons following pre‐incubation with TMRM (red). GFP expression is also visualized (green). Mitochondria appear aggregated and dysmorphic in 122 CAG cells, compared with the reticular appearance of mitochondria in 30 CAG cells (yellow arrows). Scale bar = 20 µm. B, There is a trend towards a reduced basal Ψm in 71 CAG cells as measured by confocal live imaging with TMRM but not in the 122 CAG line. Mean intensities per unit volume were calculated from 3 to 4 non‐overlapping FOVs from three independent cultures per line, and calculated as a percentage of control (30 CAG). Data are shown as mean ± SEM. C, Individual mitochondria detected within cells were classified as small (<100 µm^3^), medium (100‐1000 µm^3^), or large (>1000 µm^3^), and the sum of all mitochondrial volumes within each category expressed as a percentage of total mitochondrial volume. There is an apparent reduction in the proportion of small mitochondria and increase in proportion of medium‐sized mitochondrial puncta in 122 CAG neurons. D, Quantification of OPA1 isoforms by band densitometry of the Western blot from 30 CAG and 122 CAG DD14 samples showed no significant difference in expression of long‐form, short‐form, or total OPA1 normalized to GAPDH, or the ratio of long and short forms. Data are shown as mean ± SEM

We next assessed a potential knock‐on effect of reduced ETC activity to basal mitochondrial potential (Ψm) by measuring uptake of the membrane potential‐dependent cationic dye, TMRM (Figure 7A,B and Figure S5A). Surprisingly this was not compromised in the HD lines; in fact there was a trend to increased mean TMRM intensity in the line showing respiratory impairment, suggesting a compensatory reversal of the ATP‐synthase (complex V) observed in other models and thought to prevent Ψm collapse by reversing the pumping of H + ions.^31^ TMRM imaging also revealed potential differences in mitochondrial morphology in the HD lines; whereas the 30 CAG and GFP only (not shown) lines had well‐distributed, filamentous mitochondria, the 71 and 122 CAG neurons contained mitochondria that were irregularly distributed, clumped or enlarged (Figure 7A, arrows). To attempt to quantify this objectively, we compared the individual volumes of TMRM positive organelles generated as part of the TMRM assay. The sizes of mitochondrial puncta varied from ~1 to >1000 µm^3^ and we analyzed the relative proportions of mitochondria with different volumes within the exon 1 *HTT* lines (Figure 7C **)**. Specifically, there was an increase in the percentage of total mitochondrial volume that was comprised of medium size mitochondria (100‐1000 µm^3^) in the 122 CAG neurons, compared to the 71 CAG by one‐way ANOVA (*P* = .0363). We speculated that there may be an imbalance in the fusion‐fission processes caused by exon 1 HTT expression. Therefore, Western blotting was performed on the control (30 CAG) vs longest length 122 CAG neuronal samples to quantify levels of the pro‐fusion GTPase OPA1 (Figure S5B). We found that the expression of the short form, long form, total or the ratio of long/short form of OPA1, although trending towards an increase in the 122 CAG neurons, were not significantly different (Figure 7D).

## 4 DISCUSSION

We have generated a human neural stem cell line that expresses exon 1 *HTT* with 30, 71, and 122 CAGs at relatively matched expression levels on an isogenic background, as well as a negative control line expressing only GFP. This allelic series generates cultures containing a very high proportion of βIII‐tubulin positive neurons after a simple two‐week differentiation protocol. In contrast to some other cells lines that express mutant exon 1 HTT, these lines do not show any overt cell death or toxicity at up to six weeks in culture, though there is a possible subtle deficit in neuronal differentiation in the 122 CAG line. They do show, however, CAG‐length dependent, “pre‐pathological” changes in terms of IB formation, increased nuclear size, and mitochondrial dysfunction. These cellular changes may reflect some of the earliest changes that occur in HD pathogenesis prior to disease onset.

Multiple lines of evidence highlight the importance of exon 1 HTT in the pathogenesis of HD,^3^, ^5^, ^32^ and a mechanism for generation of mutant exon 1 HTT has been demonstrated in mouse models^7^ and patient‐derived fibroblasts and post‐mortem human brain tissue.^8^ Whilst some cell models have demonstrated rapid onset of cell toxicity and cell death on expression of exon 1 mutant HTT,^9^, ^11^, ^33^ many others do not.^34^, ^35^ For example, a study by Kim *et al* 2016 also did not find any overt cell death at baseline in an exon 1 HTT cell model as assessed by LDH assay and MTT assay, despite finding polyQ‐length‐dependent changes in protein interactions.^35^ The recapitulation of the inclusion formation that is pathognomic of HD confirms our model's validity as an HD cell model.

Many neurodegenerative diseases have protein aggregates as a cardinal feature, for example amyloid inclusions derived from amyloid precursor protein in Alzheimer's disease and alpha‐synuclein in Parkinson's disease. In HD, mutant HTT positive inclusions are found in the nuclei and neurites of patient derived brain tissue.^3^ Inclusion frequency and rate of formation are polyQ length‐dependent and inclusion size increases with disease duration,^36^, ^37^ as also demonstrated by our model system. The definitive role of these inclusions (whether toxic or protective) has not yet been established. Toxicity is suggested by experiments showing that the reduction of aggregate formation in mice correlated with improvements in phenotype.^38^, ^39^ Inclusions are thought to sequester and decrease levels of essential cellular proteins such as transcription factors,^40^ chaperones,^41^ and nuclear‐cytoplasmic transport machinery,^42^ and mutant HTT aggregates can also physically obstruct subcellular transport due to their large size.^43^ The toxic effects of mutant HTT aggregates may cause neuronal dysfunction without necessarily leading to cell death and distinct structural forms of HTT aggregates may differ in terms of their relative contribution to cell toxicity.^44^ In our model system, the presence of IB formation was not associated with cell death and those cells with inclusion‐containing nuclei did not display abnormal shaped or fragmented nuclei, even though they were significantly larger. It was not possible to measure total cytoplasmic area of IB‐containing cells due to the complex and overlapping nature of neuronal cultures, but this may also have been compromised and warrants further investigation. By way of explanation, it is possible that the increased nuclear size in the IB containing cells is a spatial effect of the inclusions themselves, or may be due to an osmotic effect, drawing water into the nuclei and expanding their size. This may lead in turn to impaired cell function, including aspects of the transcriptional dysregulation that is known to occur in HD, or due to disrupted nucleocytoplasmic transport.^45^ Instead, we observed that cells with inclusions frequently showed reduced staining intensity of cell markers in the perinuclear region, potentially reflective of transcriptional downregulation at least of these genes. This is supported by recent findings showing that the formation of HTT IBs triggers a state of cellular quiescence, as a result of co‐aggregation with other proteins.^46^

Cellular respiratory deficits have been reported previously in HD cell models, including primary cultures derived from mouse R6/2 and rat BACHD models,^47^, ^48^, ^49^, ^50^ but the effects were inconsistent. Other reports using the methods outlined here show no differences in OCR or ATP production in isolated rodent neurons.^51^ Data obtained so far from human (HD patient‐derived) cells, however, have consistently shown impaired respiration compared with controls.^52^, ^53^ Our aim in assessing the respiratory function of exon 1 HTT expressing human neuronal cells was to help resolve whether respiratory impairment is a feature of mutant HTT cytotoxicity. Our findings are in line with previous reports of impaired basal respiration, maximal respiration, and ATP production in human HD cell models. Furthermore, this toxic effect can be attributed to the mutant exon 1 HTT fragment, which exerts its pathological effect at higher polyQ lengths, even in neural stem cells, and is not restricted to those cells in which nuclear inclusions have formed. In fact, as the percentage of cells which go on to develop detectable aggregates is very small, these cells could not by any reasonable assumption be contributing to the overall mitochondrial impairment of the cultures seen in neurons, thereby the mutant exon 1 HTT‐dependent effect on respiration and ATP levels must be attributable to HTT not present in nuclear aggregates. Reduced levels and/or activity of electron transport chain components is a well‐known phenomenon in HD.^28^ Again, we confirm this significant biochemical defect in our cell model; specifically the reduction of in vitro enzymatic activity of complex I and III in exon 1 HTT 122 CAG neurons, with a clear effect of increasing CAG length on electron transport chain impairment. Our model demonstrates that the exon 1 HTT fragment is sufficient to cause mitochondrial dysfunction, by a mechanism that is incompletely defined but likely is not via altered expression of respiratory chain complexes. The primary knock‐on effects of respiratory chain dysfunction are arguably reduced ATP production, which in turn will affect an array of intracellular metabolic processes, but for neurons with an exceptionally high metabolic demand, even small reductions in ATP availability could contribute to compromised functionality, for example, in ionic homeostasis and resultant excitotoxicity.^54^, ^55^ The altered morphology of mitochondria within our HD lines was notable and could represent either an imbalance of fusion‐fission dynamics or disrupted organellar transport. Mitochondrial dysmorphology and increased fission is increasingly being reported in HD models.^56^ We did not find any significant alteration in the expression levels of long or short isoforms of the GTPase OPA1 in the HD lines tested, suggesting this was not due to disruption to pro‐fusion components.

Studying in vitro cell models of HD is critical for understanding of cell‐level effects of mutant HTT on individual cell function and behaviour that is difficult to study in whole organisms. Due to the ongoing, urgent need to develop novel therapeutics for HD, there is also demand for robust cellular models for high throughput screening of pre‐clinical compounds. Models expressing full‐length mutant HTT may more closely reflect the actual cellular physiology of HD, but N‐terminal or exon 1 models give rise to disease phenotypes, for example inclusion formation, on a time‐scale more compatible with in vitro experiments. Our model is from a human source (HD is a disease that affects only humans), and reflects the primary site of pathology in HD (neurons). On a practical level, pluripotent stem cell‐derived neurons take weeks or months to generate, and so far have generally not recapitulated the inclusion pathology that is characteristic of HD.^57^ Neurons derived from human embryonic stem cells (hESCs) expressing the mutant HTT exon 1 transgene (Q73 and Q145) display HTT inclusions only after several months.^58^ Our “intermediate model” has the benefits, as demonstrated here, of accelerated pathology, ease of rapid differentiation, quick turnover time, and amenability to high throughput formats that are useful in a screening context.

## CONFLICT OF INTEREST

Through the offices of UCL Consultants Ltd, a wholly owned subsidiary of University College London, S.J.Tabrizi has also undertaken consultancy services for Takeda Pharmaceuticals Ltd. No other author has any conflict of interest to declare.

## AUTHOR CONTRIBUTIONS

R. Ghosh, A. Wood‐Kaczmar, R. Andre, S.J. Heales, I.P. Hargreaves, and S.J. Tabrizi designed the research; R. Ghosh, A. Wood‐Kaczmar, L. Dobson, F. Herrmann, I.P. Hargreaves, R. Heaton, and A.J. Lam performed the research; E.C. Siranathsinghji, E.J. Smith, G.P. Bates, R. Ketteler, and A.Y. Abramov contributed new reagents/analytic tools; R. Ghosh, A. Wood‐Kaczmar, R. Andre, and J. Kriston‐Vizi analyzed the data; R. Ghosh, A. Wood‐Kaczmar, and R. Andre wrote the first draft of the manuscript. All authors commented on the manuscript.

## Supporting information

Fig S1‐S5

## References

[1] Ross CA, Tabrizi SJ. Huntington's disease: from molecular pathogenesis to clinical treatment. Lancet Neurol. 2011;10:83‐98. 21163446 10.1016/S1474-4422(10)70245-3

[2] Andrew SE, Paul Goldberg Y, Kremer B, et al. The relationship between trinucleotide (CAG) repeat length and clinical features of Huntington's disease. Nat Genet. 1993;4:398‐403. 8401589 10.1038/ng0893-398

[3] DiFiglia M, Sapp E, Chase KO, et al. Aggregation of huntingtin in neuronal intranuclear inclusions and dystrophic neurites in brain. Science. 1997;277:1990‐1993. 9302293 10.1126/science.277.5334.1990

[4] Gutekunst CA, Li SH, Yi H, et al. Nuclear and neuropil aggregates in Huntington's disease: relationship to neuropathology. J Neurosci. 1999;19:2522‐2534. 10087066 10.1523/JNEUROSCI.19-07-02522.1999 PMC6786077

[5] Mangiarini L, Sathasivam K, Seller M, et al. Exon 1 of the HD gene with an expanded CAG repeat is sufficient to cause a progressive neurological phenotype in transgenic mice. Cell. 1996;87:493‐506. 8898202 10.1016/s0092-8674(00)81369-0

[6] Barbaro BA, Lukacsovich T, Agrawal N, et al. Comparative study of naturally occurring huntingtin fragments in Drosophila points to exon 1 as the most pathogenic species in Huntington's disease. Hum Mol Genet. 2015;24:913‐925. 25305076 10.1093/hmg/ddu504 PMC4834878

[7] Sathasivam K, Neueder A, Gipson TA, et al. Aberrant splicing of HTT generates the pathogenic exon 1 protein in Huntington disease. Proc Natl Acad Sci U S A. 2013;110:2366‐2370. 23341618 10.1073/pnas.1221891110 PMC3568346

[8] Neueder A, Landles C, Ghosh R, et al. The pathogenic exon 1 HTT protein is produced by incomplete splicing in Huntington's disease patients. Sci Rep. 2017;7:1‐10. 28465506 10.1038/s41598-017-01510-z PMC5431000

[9] Ho LW, Brown R, Maxwell M, Wyttenbach A, Rubinsztein DC. Wild type Huntingtin reduces the cellular toxicity of mutant Huntingtin in mammalian cell models of Huntington's disease. J Med Genet. 2001;38:450‐452. 11432963 10.1136/jmg.38.7.450 PMC1757193

[10] Ye C, Zhang Y, Wang W, Wang J, Li H. Inhibition of neurite outgrowth and promotion of cell death by cytoplasmic soluble mutant huntingtin stably transfected in mouse neuroblastoma cells. Neurosci Lett. 2008;442:63‐68. 18650014 10.1016/j.neulet.2008.05.119

[11] Sahoo B, Arduini I, Drombosky KW, et al. Folding landscape of mutant Huntingtin exon1: diffusible multimers, oligomers and fibrils, and no detectable monomer. PLoS ONE. 2016;11:e0155747. 27271685 10.1371/journal.pone.0155747 PMC4894636

[12] Germain PL, Testa G. Taming human genetic variability: transcriptomic meta‐analysis guides the experimental design and interpretation of iPSC‐based disease modeling. Stem Cell Reports. 2017;8:1784‐1796. 28591656 10.1016/j.stemcr.2017.05.012 PMC5470233

[13] Donato R, Miljan EA, Hines SJ, et al. Differential development of neuronal physiological responsiveness in two human neural stem cell lines. BMC Neurosci. 2007;8:36. 17531091 10.1186/1471-2202-8-36 PMC1888696

[14] Wood‐Kaczmar A, Gandhi S, Yao Z, et al. PINK1 is necessary for long term survival and mitochondrial function in human dopaminergic neurons. PLoS ONE. 2008;3:e2455. 18560593 10.1371/journal.pone.0002455 PMC2413012

[15] Antoniou M, Harland L, Mustoe T, et al. Transgenes encompassing dual‐promoter CpG islands from the human TBP and HNRPA2B1 loci are resistant to heterochromatin‐mediated silencing. Genomics. 2003;82:269‐279. 12906852 10.1016/s0888-7543(03)00107-1

[16] Bayram‐Weston Z, Jones L, Dunnett SB, Brooks SP. Light and electron microscopic characterization of the evolution of cellular pathology in the R6/1 Huntington's disease transgenic mice. Brain Res Bull. 2012;88:104‐112. 21801812 10.1016/j.brainresbull.2011.07.009

[17] Macdonald D, Tessari MA, Boogaard I, et al. Quantification assays for total and polyglutamine‐expanded huntingtin proteins. PLoS ONE. 2014;9:e96854. 24816435 10.1371/journal.pone.0096854 PMC4016121

[18] Ko J, Isas JM, Sabbaugh A, et al. Identification of distinct conformations associated with monomers and fibril assemblies of mutant huntingtin. Hum Mol Genet. 2018;27:2330–2343. 29912367 10.1093/hmg/ddy141 PMC6005051

[19] Schneider CA, Rasband WS, Eliceiri KW. NIH Image to ImageJ: 25 years of image analysis. Nat Methods. 2012;9:671‐675. 22930834 10.1038/nmeth.2089 PMC5554542

[20] R Core Development Team . R: A Language and Environment for Statistical Computing. Vienna, Austria: R Foundation for Statistical Computing; 2018.

[21] Hargreaves IP, Heales SJ, Land JM. Mitochondrial respiratory chain defects are not accompanied by an increase in the activities of lactate dehydrogenase or manganese superoxide dismutase in paediatric skeletal muscle biopsies. J Inherit Metab Dis. 1999;22:925‐931. 10604144 10.1023/a:1005643508075

[22] King TS. Preparations of succinate—cytochrome c reductase and the cytochrome b*‐*c 1 particle, and reconstitution of succinate‐cytochrome c reductase. Methods Enzymol. 1967;10:216‐225.

[23] Hatefi Y, Stiggall DL. Preparation and properties of succinate: ubiquinone oxidoreductase (complex II). Methods Enzymol. 1978;53:21‐27. 713835 10.1016/s0076-6879(78)53008-5

[24] Kirby DM, Thorburn DR, Turnbull DM, Taylor RW. Biochemical assays of respiratory chain complex activity. In: Pon LA, Schon EA, eds. Methods in Cell Biology. Vol. 80. Amsterdam, the Netherlands: Elsevier; 2007: 93–119. 10.1016/S0091-679X(06)80004-X 17445690

[25] Duncan AJ, Heales SJ, Mills K, Eaton S, Land JM, Hargreaves IP. Determination of coenzyme Q10 status in blood mononuclear cells, skeletal muscle, and plasma by HPLC with di‐propoxy‐coenzyme Q10 as an internal standard. Clin Chem. 2005;51:2380‐2382. 16306103 10.1373/clinchem.2005.054643

[26] Selak MA, de Chadarevian JP, Melvin JJ, Grover WD, Salganicoff L, Kaye EM. Mitochondrial activity in Pompe's disease. Pediatr Neurol. 2000;23:54‐57. 10963971 10.1016/s0887-8994(00)00145-4

[27] Kim YH, Choi SH, D'Avanzo C, et al. A 3D human neural cell culture system for modeling Alzheimer's disease. Nat Protoc. 2015;10:985‐1006. 26068894 10.1038/nprot.2015.065 PMC4499058

[28] Polyzos AA, McMurray CT. The chicken or the egg: mitochondrial dysfunction as a cause or consequence of toxicity in Huntington's disease. Mech Ageing Dev. 2017;161:181‐197. 27634555 10.1016/j.mad.2016.09.003 PMC5543717

[29] Rahman S, Hargreaves I, Clayton P, Heales S. Neonatal presentation of coenzyme Q10 deficiency. J Pediatr. 2001;139:456‐458. 11562630 10.1067/mpd.2001.117575

[30] López LC, Quinzii CM, Area E, et al. Treatment of CoQ(10) deficient fibroblasts with ubiquinone, CoQ analogs, and vitamin C: time‐ and compound‐dependent effects. PLoS ONE. 2010;5:e11897. 20689595 10.1371/journal.pone.0011897 PMC2912846

[31] Gandhi S, Wood‐Kaczmar A, Yao Z, et al. PINK1‐associated Parkinson's disease is caused by neuronal vulnerability to calcium‐induced cell death. Mol Cell. 2009;33:627‐638. 19285945 10.1016/j.molcel.2009.02.013 PMC2724101

[32] Yang SH, Cheng PH, Banta H, et al. Towards a transgenic model of Huntington's disease in a non‐human primate. Nature. 2008;453:921‐924. 18488016 10.1038/nature06975 PMC2652570

[33] Miller J, Arrasate M, Shaby BA, Mitra S, Masliah E, Finkbeiner S. Quantitative relationships between huntingtin levels, polyglutamine length, inclusion body formation, and neuronal death provide novel insight into huntington's disease molecular pathogenesis. J Neurosci. 2010;30:10541‐10550. 20685997 10.1523/JNEUROSCI.0146-10.2010 PMC3078518

[34] Apostol BL, Kazantsev A, Raffioni S, et al. A cell‐based assay for aggregation inhibitors as therapeutics of polyglutamine‐repeat disease and validation in Drosophila. Proc Natl Acad Sci U S A. 2003;100:5950‐5955. 12730384 10.1073/pnas.2628045100 PMC156307

[35] Kim YE, Hosp F, Frottin F, et al. Soluble oligomers of polyQ‐expanded Huntingtin target a multiplicity of key cellular factors. Mol Cell. 2016;63:951‐964. 27570076 10.1016/j.molcel.2016.07.022

[36] Hughes A, Jones L. Pathogenic mechanisms in Huntington's disease. In: Bates GP, Tabrizi SJ, Jones L, eds. Huntington's Disease. Oxford: Oxford University Press; 2014.

[37] Scherzinger E, Sittler A, Schweiger K, et al. Self‐assembly of polyglutamine‐containing huntingtin fragments into amyloid‐like fibrils: implications for Huntington's disease pathology. Proc Natl Acad Sci U S A. 1999;96:4604‐4609. 10200309 10.1073/pnas.96.8.4604 PMC16379

[38] Labbadia J, Cunliffe H, Weiss A, et al. Altered chromatin architecture underlies progressive impairment of the heat shock response in mouse models of Huntington disease. J Clin Invest. 2011;121:3306‐3319. 21785217 10.1172/JCI57413 PMC3148745

[39] Labbadia J, Novoselov SS, Bett JS, et al. Suppression of protein aggregation by chaperone modification of high molecular weight complexes. Brain. 2012;135:1180‐1196. 22396390 10.1093/brain/aws022 PMC3326252

[40] Schaffar G, Breuer P, Boteva R, et al. Cellular toxicity of polyglutamine expansion proteins: mechanism of transcription factor deactivation. Mol Cell. 2004;15:95‐105. 15225551 10.1016/j.molcel.2004.06.029

[41] Park SH, Kukushkin Y, Gupta R, et al. PolyQ proteins interfere with nuclear degradation of cytosolic proteins by sequestering the Sis1p chaperone. Cell. 2013;154:134‐145. 23791384 10.1016/j.cell.2013.06.003

[42] Woerner AC, Frottin F, Hornburg D, et al. Cytoplasmic protein aggregates interfere with nucleocytoplasmic transport of protein and RNA. Science. 2016;351:173‐176. 26634439 10.1126/science.aad2033

[43] Li H, Wyman T, Yu ZX, Li SH, Li XJ. Abnormal association of mutant huntingtin with synaptic vesicles inhibits glutamate release. Hum Mol Genet. 2003;12:2021‐2030. 12913073 10.1093/hmg/ddg218

[44] Nekooki‐Machida Y, Kurosawa M, Nukina N, Ito K, Oda T, Tanaka M. Distinct conformations of in vitro and in vivo amyloids of huntingtin‐exon1 show different cytotoxicity. Proc Natl Acad Sci U S A. 2009;106:9679‐9684. 19487684 10.1073/pnas.0812083106 PMC2689308

[45] Grima JC, Daigle JG, Arbez N, et al. Mutant Huntingtin disrupts the nuclear pore complex. Neuron. 2017;94:93‐107.e106. 28384479 10.1016/j.neuron.2017.03.023 PMC5595097

[46] Ramdzan YM, Trubetskov MM, Ormsby AR, et al. Huntingtin inclusions trigger cellular quiescence, deactivate apoptosis, and lead to delayed necrosis. Cell Rep. 2017;19:919‐927. 28467905 10.1016/j.celrep.2017.04.029

[47] Lou S, Lepak VC, Eberly LE, et al. Oxygen consumption deficit in Huntington disease mouse brain under metabolic stress. Hum Mol Genet. 2016;25:2813‐2826. 27193167 10.1093/hmg/ddw138 PMC5181641

[48] Ismailoglu I, Chen Q, Popowski M, Yang L, Gross SS, Brivanlou AH. Huntingtin protein is essential for mitochondrial metabolism, bioenergetics and structure in murine embryonic stem cells. Dev Biol. 2014;391:230‐240. 24780625 10.1016/j.ydbio.2014.04.005 PMC4109978

[49] Clemens LE, Weber JJ, Wlodkowski TT, et al. Olesoxime suppresses calpain activation and mutant huntingtin fragmentation in the BACHD rat. Brain. 2015;138:3632‐3653. 26490331 10.1093/brain/awv290

[50] Gouarné C, Tardif G, Tracz J, et al. Early deficits in glycolysis are specific to striatal neurons from a rat model of Huntington disease. PLoS ONE. 2013;8:e81528. 24303051 10.1371/journal.pone.0081528 PMC3841140

[51] Hamilton J, Pellman JJ, Brustovetsky T, Harris RA, Brustovetsky N. Oxidative metabolism in YAC128 mouse model of Huntington's disease. Hum Mol Genet. 2015;24:4862‐4878. 26041817 10.1093/hmg/ddv209 PMC4527489

[52] Mejia EM, Chau S, Sparagna GC, Sipione S, Hatch GM. Reduced mitochondrial function in human Huntington disease lymphoblasts is not due to alterations in cardiolipin metabolism or mitochondrial supercomplex assembly. Lipids. 2016;51:561‐569. 26846325 10.1007/s11745-015-4110-0

[53] An MC, Zhang N, Scott G, et al. Genetic correction of Huntington's disease phenotypes in induced pluripotent stem cells. Cell Stem Cell. 2012;11:253‐263. 22748967 10.1016/j.stem.2012.04.026 PMC3608272

[54] Jacquard C, Trioulier Y, Cosker F, et al. Brain mitochondrial defects amplify intracellular [Ca2+] rise and neurodegeneration but not Ca2+ entry during NMDA receptor activation. FASEB J. 2006;20:1021‐1023. 16571773 10.1096/fj.05-5085fje

[55] Simpson JR, Isacson O. Mitochondrial impairment reduces the threshold for in vivo NMDA‐mediated neuronal death in the striatum. Exp Neurol. 1993;121:57‐64. 8495711 10.1006/exnr.1993.1071

[56] Guedes‐Dias P, Pinho BR, Soares TR, de Proença J, Duchen MR, Oliveira JM. Mitochondrial dynamics and quality control in Huntington's disease. Neurobiol Dis. 2016;90:51‐57. 26388396 10.1016/j.nbd.2015.09.008

[57] The Hd Ipsc Consortium . Induced pluripotent stem cells from patients with Huntington's disease show CAG‐repeat‐expansion‐associated phenotypes. Cell Stem Cell. 2012;11:264‐278. 22748968 10.1016/j.stem.2012.04.027 PMC3804072

[58] Lu B, Palacino J. A novel human embryonic stem cell‐derived Huntington's disease neuronal model exhibits mutant huntingtin (mHTT) aggregates and soluble mHTT‐dependent neurodegeneration. FASEB J. 2013;27:1820‐1829. 23325320 10.1096/fj.12-219220

