# Supplementary material for: Expression of mutant exon 1 huntingtin fragments in human neural stem cells and neurons causes inclusion formation and mitochondrial dysfunction
Source: FASEB J. 2020 Apr 23;34(6):8139–54. doi: 10.1096/fj.201902277RR (PMC8432155; doi:10.1096/fj.201902277RR)
Supplement: Supplementary file 1 — Fig S1‐S5 [file FSB2-34-8139-s001.pdf]

# Supplemental Figures

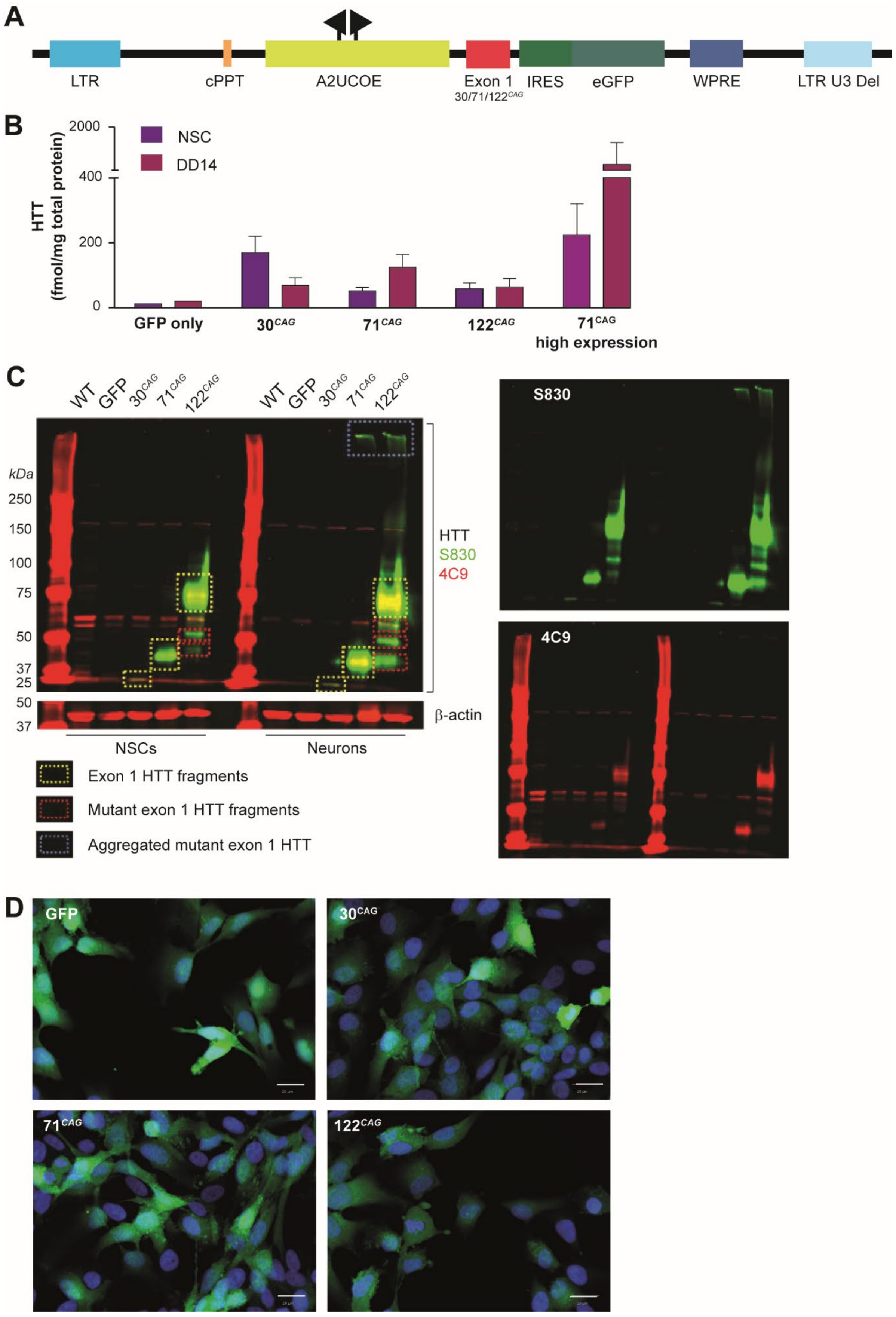

**Supplemental Figure S1. The generation of ReNcell VM exon 1 HTT lines. A)** Schematic representation of the lentiviral vector used to create the exon 1 HTT expressing lines. (LTR = long terminal repeat; cPPT = central polyproline tract; UCOE = ubiquitous chromatin opening element; IRES = internal ribosome entry site; eGFP = enhanced green fluorescent protein; WPRE = Woodchuck hepatitis virus post-transcriptional regulatory element.) **B)** Total HTT levels across the ReNcell VM exon 1 HTT panel in NSCs and following two weeks' neuronal differentiation (DD14), as determined by multiplex ELISA using a combination of anti-HTT antibodies (2B7 and 4C9). The 71 CAG high expression line was used as a positive control. Whilst each of the transduced lines showed HTT over-expression, there were no consistent *HTT* CAG or differentiation-dependent differences; data shown as mean ± SEM. **C)** Western blot of the ReNcell VM exon 1 HTT lines showing expression of exon 1 HTT fragments in each in both their NSC and DD14 neuronal states. Samples were run on an 8% tris-glycine gel and the stacking gel retained during the Western blot; insoluble aggregated material in the stacking gel was recognised by anti-HTT antibodies (dotted blue box) in the samples obtained from neurons expressing pathogenic *HTT* CAG repeat lengths. **D)** Confocal fluorescence images showing GFP expression in each of the exon 1 HTT expressing lines in their NSC state; scale bars = 20 μm.

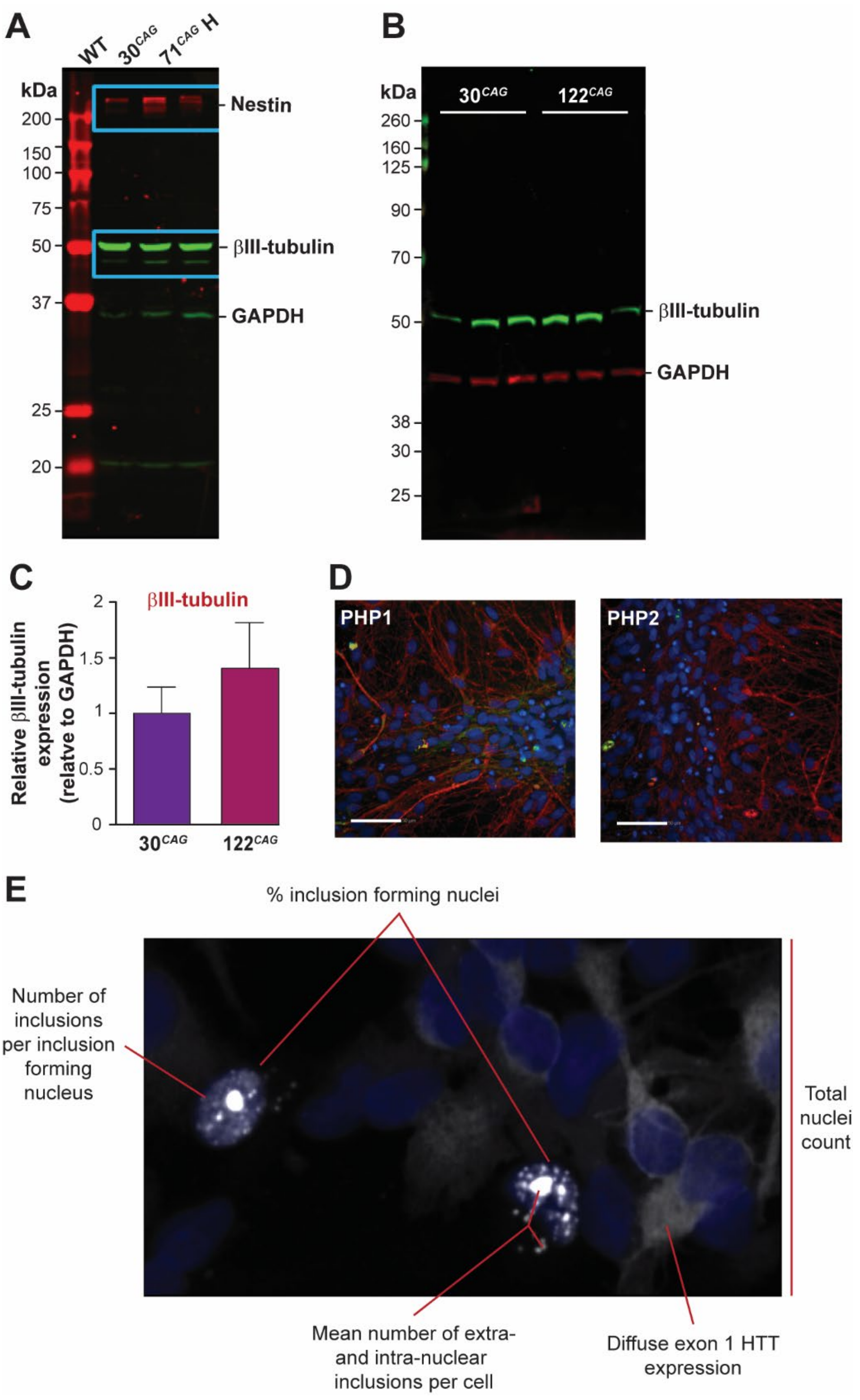


**Supplemental Figure S2. Quantification of βIII-tubulin expression and mHTT aggregates in ReNcell VM exon 1 HTT lines A)** Western blotting confirmed the expression of βIII-tubulin in 30 CAG and 71 CAG high expression ReNcell VM neurons (DD14), similar to the non-transduced (WT) parent line. Persistent low level nestin expression was also observed, as is typical of newly differentiated neurons. **B)** Western blotting for βIII-tubulin and GAPDH in multiple 30 CAG and

122 CAG neuron (DD14) cultures. When quantified, **C**) βIII-tubulin expression levels (normalised to those of GAPDH) did not differ between the 30 CAG and 122 CAG lines (n=3); data shown as mean ± SEM. **D)** Co-staining of HTT positive IBs within 71 CAG neurons (DD14) with PHP1 and PHP2 (red = S830 anti-HTT; green = PHP; blue = nuclei); scale bars = 50 μm. **E)** Cellular features quantified using HCI and ImageJ. Example field of view image taken of the differentiated (DD14) 122 CAG line, fixed and stained with S830 anti-HTT antibody.

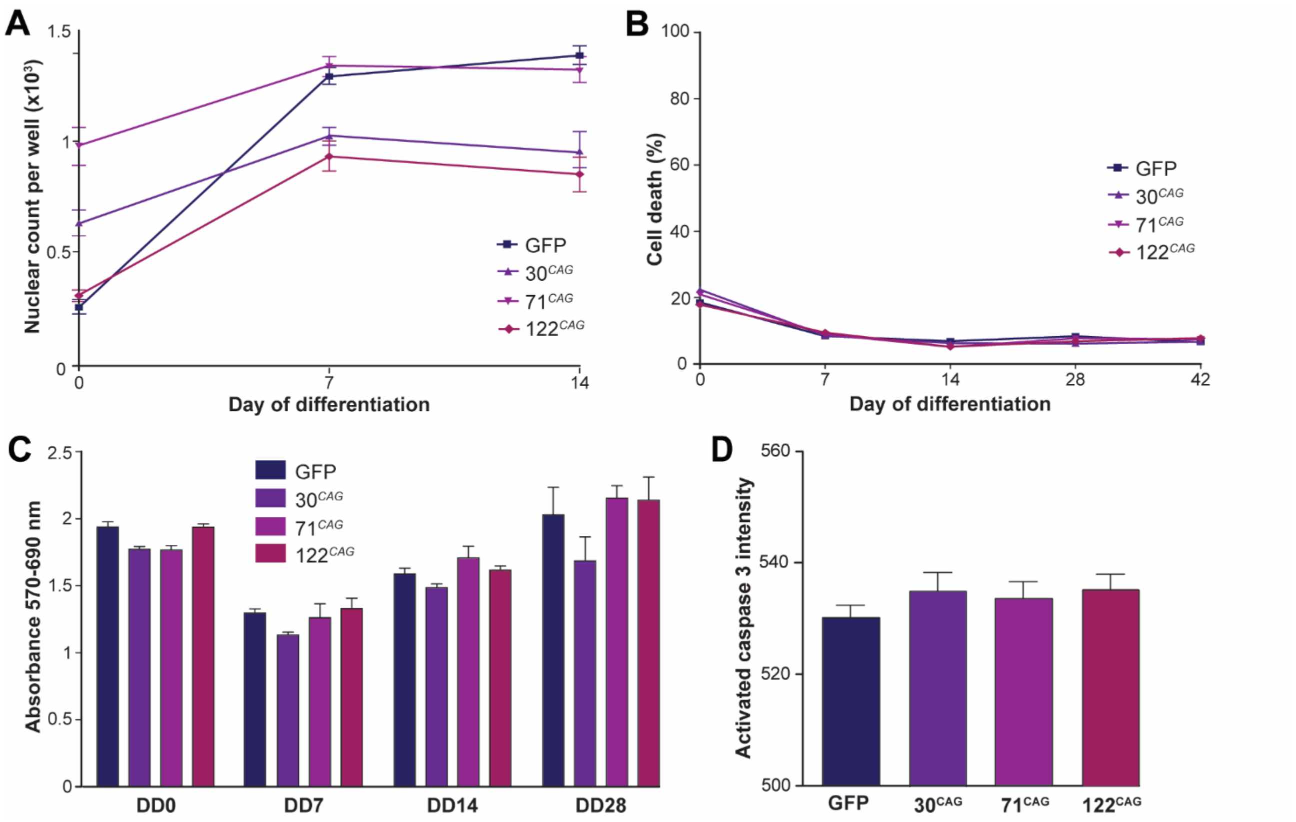


**Supplemental Figure S3. No differences in long-term cell viability between the ReNcell VM exon 1 HTT lines. A)** Total nuclear counts over time did not differ between any of the ReNcell VM exon 1 HTT lines or the GFP control (n=3). **B)** LDH assays of ReNcell VM exon 1 HTT lines differentiated up to DD42 revealed no overt cell death in any of the lines and no significant differences between them (n=8). **C)** MTT assays carried of the ReNcell VM exon 1 HTT panel up to DD28 showed no consistent changes or differences between the lines (n=8). **D**) ReNcell VM exon 1 HTT lines analysed by HCI at DD14 showed no differences in mean intensity per unit area of anti-activated caspase 3 staining (n=16). Data shown as mean ± SEM.

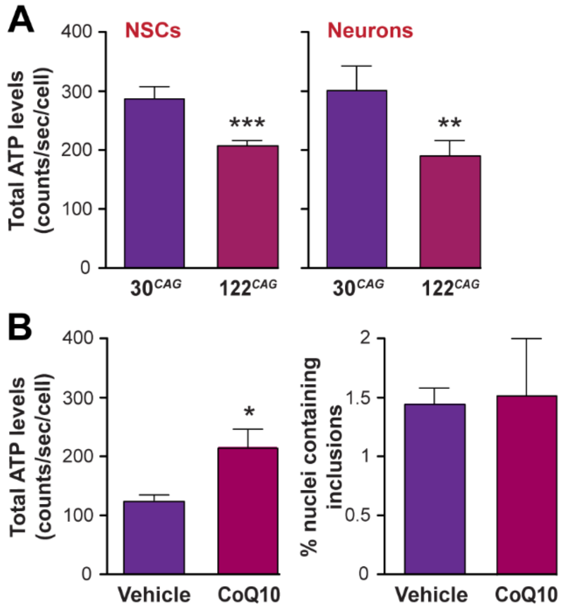


**Supplemental Figure S4. Total ATP levels that are reduced in ReNcell VM 122 CAG neurons are increased by supplementation with exogenous $CoQ_{10}$.** **A**) Total ATP levels were reduced in both NSCs and neurons (DD7) of the ReNcell VM 122 CAG line compared to its 30 CAG counterpart (n=5-6). **B)** Total ATP levels within 122 CAG neurons (DD9) were significantly increased by two days' supplementation of culture medium with exogenous $CoQ_{10}$ (5 µM) when compared to the ethanol vehicle alone (n=5-6). The percentage of nuclei containing mHTT IBs at DD14, however, was not significantly altered by supplementation of culture medium with exogenous $CoQ_{10}$. Data are shown as mean ± SEM; *p<0.05, ** $p$ <0.01, *** $p$< 0.001.

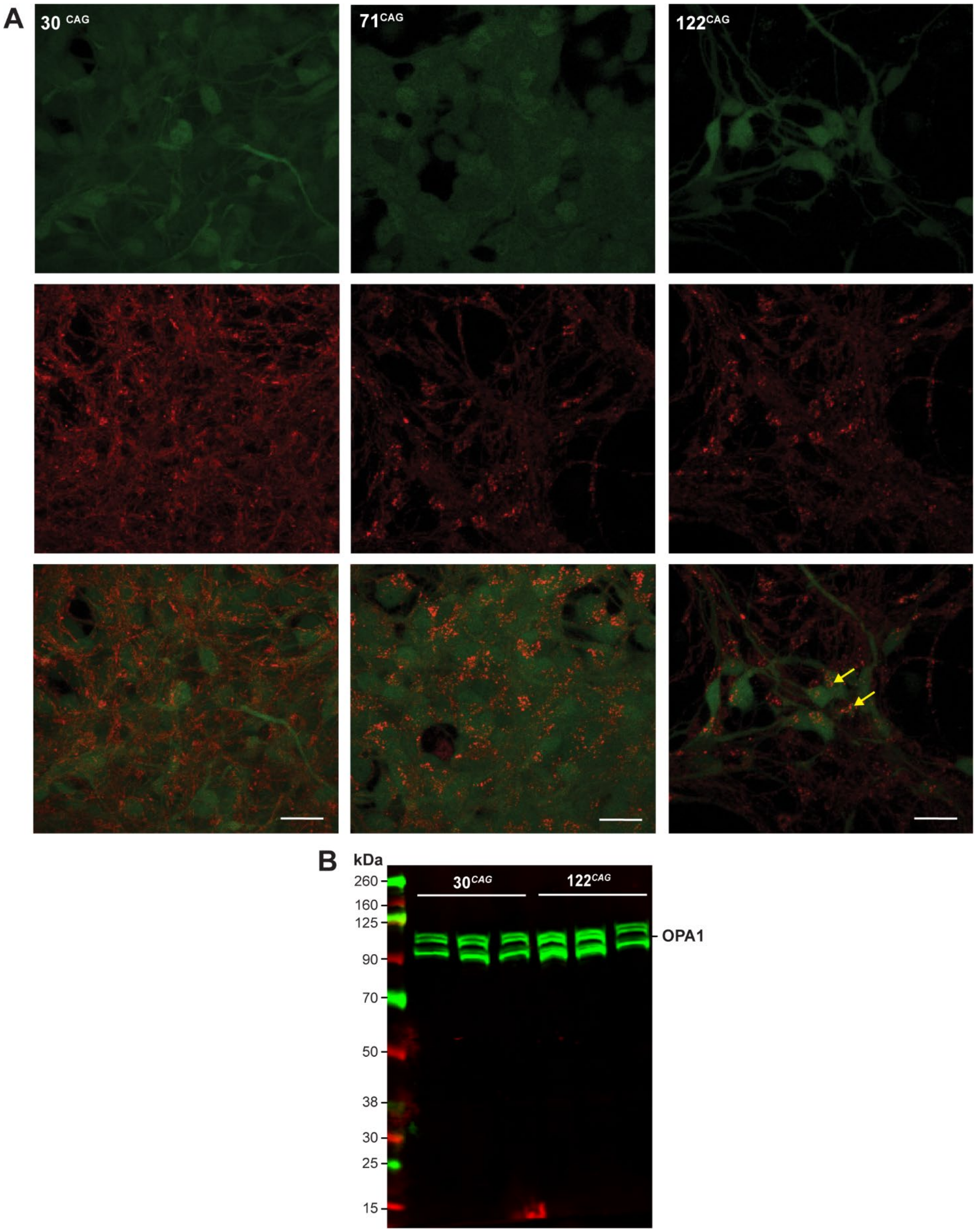


**Supplemental Figure S5. Mitochondrial morphology in ReNcell VM exon 1 HTT lines. A)** Maximum intensity projection images from separate channels or merged images of z-stacks taken during live imaging of ReNcell VM exon 1 HTT neurons following pre-incubation with TMRM

(red). GFP expression is also visualised (green); scale bar 20 = μm. **B)** Western blot of 30 CAG and 122 CAG DD14 neurons to quantify OPA1 expression (green = OPA1).
